# Recent Progress in Photocathode Interface Engineering for Photoelectrochemical CO_2_ Reduction Reaction to C_1_ or C_2+_ Products

**DOI:** 10.1002/EXP.20240014

**Published:** 2025-02-04

**Authors:** Jae Hak Kim, Sung Hyun Hong, Sang Hyun Ahn, Soo Young Kim

**Affiliations:** ^1^ Department of Materials Science and Engineering Korea University Seoul Republic of Korea; ^2^ School of Chemical Engineering and Material Science Chung‐Ang University Seoul Republic of Korea

**Keywords:** cocatalysts engineering, defect engineering, interface engineering, junction engineering, nanostructure engineering, photoelectrochemical CO_2_ reduction

## Abstract

Photoelectrochemical (PEC) systems harness light absorption to initiate chemical reactions, while electrochemical reactions facilitate the conversion of reactants into desired products, ensuring more efficient and sustainable energy conversion in PECs. Central to optimizing the performance of PECs was the pivotal role played by interface engineering. This intricate process involves manipulating material interfaces at the atomic or nanoscale to enhance charge transfer, improve catalytic activity, and address limitations associated with bulk materials. The careful tuning of factors such as band gap, surface energy, crystallinity, defect characteristics, and structural attributes through interface engineering led to superior catalytic efficiency. Specifically, interface engineering significantly enhanced the efficiency of semiconductor‐based PECs. Engineers strategically designed heterojunctions and manipulated catalyst surface properties to optimize the separation and migration of photogenerated charge carriers, minimizing recombination losses and improving performance overall. This review categorizes the discussion into four sections focusing on the interface engineering of PECs, providing valuable insights into recent research trends. Overall, the synergy between PECs and interface engineering holds tremendous promise for advancing renewable energy technologies and addressing environmental challenges by offering innovative solutions for sustainable energy conversion and storage.

## Graphical abstract

Strategic interface engineering optimizes photocathode design for enhanced photoelectrochemical CO_2_ reduction, incorporating cocatalyst engineering, junction engineering, nanostructure engineering, and defect engineering enhances performance efficiency.

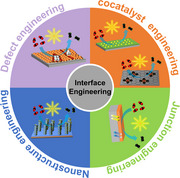

## 1 Introduction

As industrialization increases worldwide, the number of artificially generated greenhouse gases has risen, continuing to exacerbate climate change [1, 2, 3]. Efforts to reduce carbon dioxide (CO_2_), the primary contributor to this issue, are intensifying. Despite a temporary decline in global CO_2_ emissions due to the COVID‐19 pandemic in 2020, 2021 saw record‐high carbon emissions of approximately 36.1 GtCO_2_, a 6.3% increase year‐on‐year [4]. The 2021 IPCC report states that the remaining carbon budget to limit anthropogenic warming to 1.5°C and 2°C above pre‐industrial levels, starting from 2020, is 400 GtCO_2_ and 1,150 GtCO_2_, respectively [5]. Addressing climate change urgently requires innovative solutions [6].

To meet this challenge, various technologies have been developed to convert CO_2_ into industrially useful chemicals, utilizing methods such as electrochemical reduction (ECR) [7, 8, 9, 10, 11], photochemical reduction (PCR) [12, 13, 14, 15, 16], and photoelectrochemical (PEC) reduction [17, 18]. However, significant obstacles remain—CO_2_ molecules are thermodynamically stable and require considerable electrochemical overpotential for reduction. Besides, in aqueous electrolysis systems, the ECR of CO_2_ competes with the hydrogen evolution reaction (HER), necessitating the suppression of the latter to enhance the catalytic activity of the CO_2_ reduction reaction (CO_2_RR). As depicted in Table 1, ECR involves a complex reaction mechanism, producing a range of hydrocarbon compounds and potential selectivity issues [19, 20]. Product selectivity depends on the adsorption and activation energy of intermediates on the catalyst's surface. For instance, HCOOH can be selectively produced through the formation of ^*^OCHO, while ^*^COOH is a key intermediate in the formation of CO [21]. The formed ^*^COOH intermediate undergoes protonation to create a ^*^CO intermediate, which then desorbs as CO. Besides HCOOH, the reaction mechanism for other hydrocarbon products involves the ^*^CO intermediate [20, 21]. Cu's unique ability to support the formation of C_2+_ products via the two‐electron reduction of CO_2_ is due to its distinctive properties: negative adsorption energy for the ^*^CO intermediate and positive adsorption energy for the ^*^H intermediate [22]. The ^*^CO intermediate is pivotal in CO_2_ ECR, impacting C─C coupling reactions and opening pathways for C_2+_ product formation [23].

**TABLE 1:** Standard reduction potentials of the electrochemical CO_2_RR in aqueous solution at pH7.

Products	Reactions	Standard reduction potential [V vs. NHE]
Carbon monoxide (CO)	CO_2_ + 2H^+^ + 2e^–^ → CO + H_2_O	−0.53
Methane (CH_4_)	CO_2_ + 8H^+^ + 8e^–^ → CH_4_ + 2H_2_O	−0.24
Formic acid (HCOOH)	CO_2_ + 2H^+^ + 2e^–^ → HCOOH	−0.61
Methanol (CH_3_OH)	CO_2_ + 6H^+^ + 6e^–^ → CH_3_OH + H_2_O	−0.38
Ethylene (C_2_H_4_)	2CO_2_ + 12H^+^ + 12e^–^ → C_2_H_4_ + 4H_2_O	0.06
Ethanol (C_2_H_5_OH)	2CO_2_ + 12H^+^ + 12e^–^ → C_2_H_5_OH + 3H_2_O	0.08
Ethane (C_2_H_6_)	2CO_2_ + 14H^+^ + 14e^–^ → C_2_H_6_ + 4H_2_O	−0.27
Acetic acid (CH_3_COOH)	2CO_2_ + 8H^+^ + 8e^–^ → CH_3_COOH + 2H_2_O	−0.30
Isopropanol (C_3_H_7_OH)	3CO_2_ + 18H^+^ + 18e^−^ → C_3_H_7_OH + 5H_2_O	−0.31

Since the first PEC study achieved CO_2_RR using a p‐GaAs photoelectrode in 1978, sustained efforts have been made to convert photo‐driven CO_2_ into solar fuel across various fields [24]. PEC CO_2_RR has advantages over ECR, including utilizing the photovoltage generated by semiconductor photoelectrodes, reducing system complexity, and partially offsetting the electrical energy required for CO_2_RR. Also, accelerated charge separation due to bias‐induced band bending enables higher production rates than PCR. PEC cells typically comprise a photoactive semiconductor electrode (photoelectrode), an electrolyte, and a counter electrode with a metal electrode catalyst or a second photoelectrode. The CO_2_RR occurs at the cathode, while water oxidation occurs at the anode, with the photoelectrode as either or both [25, 26, 27]. In PEC, each electrode can optimize catalyst efficiency based on factors such as bandgap, surface energy, crystallinity, defect characteristics, and structural attributes. Interface engineering is a promising approach to overcoming the limitations observed in existing bulk materials.

**FIGURE 1:**
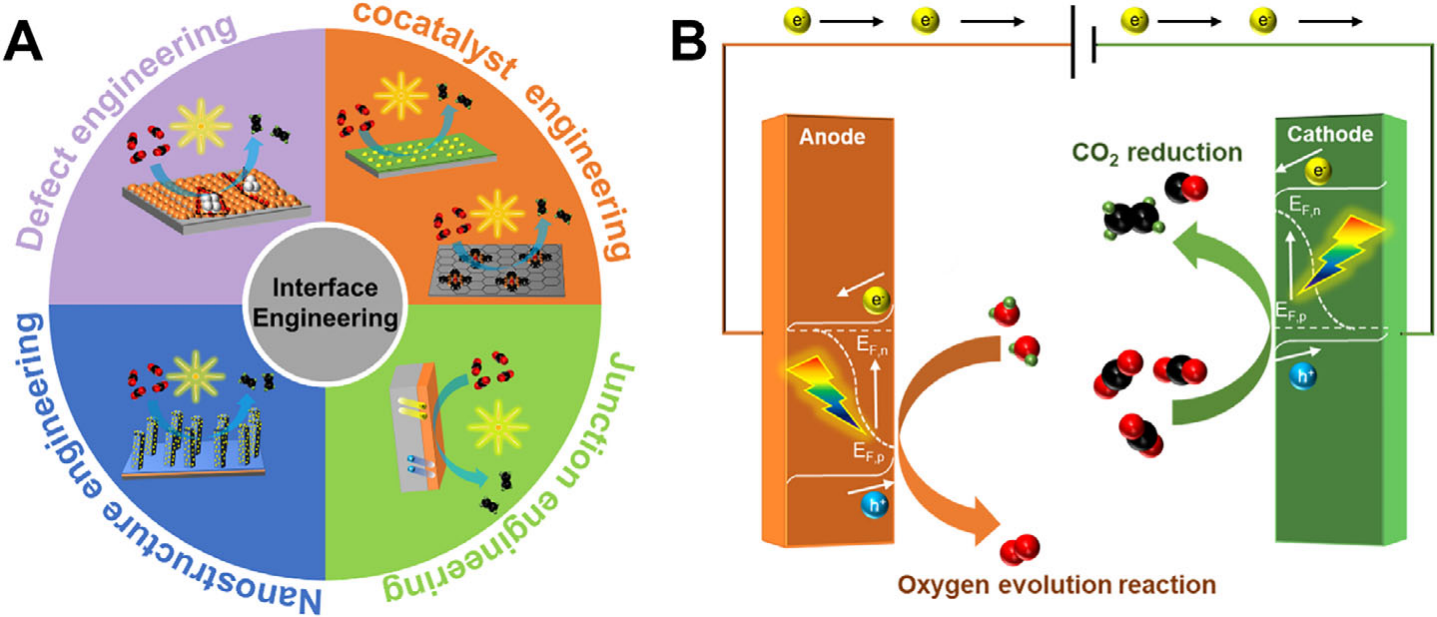
(A) Schematic of interface engineering strategies categorized into four sections. (B) Schematic illustration of basic principles of PEC CO_2_RR.

To the best of our knowledge, while several papers have addressed research developments in PEC CO_2_RR, comprehensive reviews specifically focusing on interface engineering of photocathodes for producing C_1_ or C_2+_ products in PEC CO_2_RR are still lacking. As illustrated in Figure 1A, this review is divided into four sections: cocatalyst engineering (plasmonic noble metal and non‐noble metal), junction engineering, nanostructure engineering, and defect engineering—from an interface perspective. These sections provide insights into recent research trends in this field of interface engineering of photocathodes for PEC CO_2_RR. Additionally, the objective is to compare products generated by PEC, categorizing them into C_1_ and C_2+_, and to offer insights into future research directions. Through this article, we aspire to contribute to the advancement of PEC technology grounded in interface engineering, with the ultimate goal of achieving carbon neutrality.

## 2 Basics of PEC CO_2_RR

### 2.1 Principles of PEC CO_2_RR

PEC systems offer promising avenues for CO_2_RR using solar energy [28]. In a fundamental approach, photocathodes utilizing p‐type semiconductor materials harness solar energy to generate photovoltage, serving as the primary driving force to counter the necessary potential for CO_2_RR. A standard three‐electrode PEC CO_2_RR system consists of a p‐type semiconductor photocathode for CO_2_RR, an n‐type semiconductor photoanode for the oxygen evolution reaction (OER), and a reference electrode. Immersing a p‐type semiconductor photocathode in a CO_2_‐saturated aqueous electrolyte creates a semiconductor–electrolyte interface. The difference in Fermi levels across this interface induces a built‐in electric field, causing the semiconductor's energy bands to bend downward. Upon light absorption, electron–hole pairs (e^–^/h^+^ pairs) are generated by the promotion of an electron from the semiconductor's valence band (VB) to the conduction band (CB), which then separate into free carriers. Photogenerated electrons from the photoanode migrate to the photocathode, while photogenerated holes transfer from the photocathode to the photoanode. This organized movement of electrons and holes involves the participation of electrons in the CO_2_RR and holes in the OER at the interface between the electrode and the electrolyte [19, 29]. A schematic principle of PEC CO_2_RR is depicted in Figure 1B.

### 2.2 Mechanism for CO_2_RR

**FIGURE 2:**
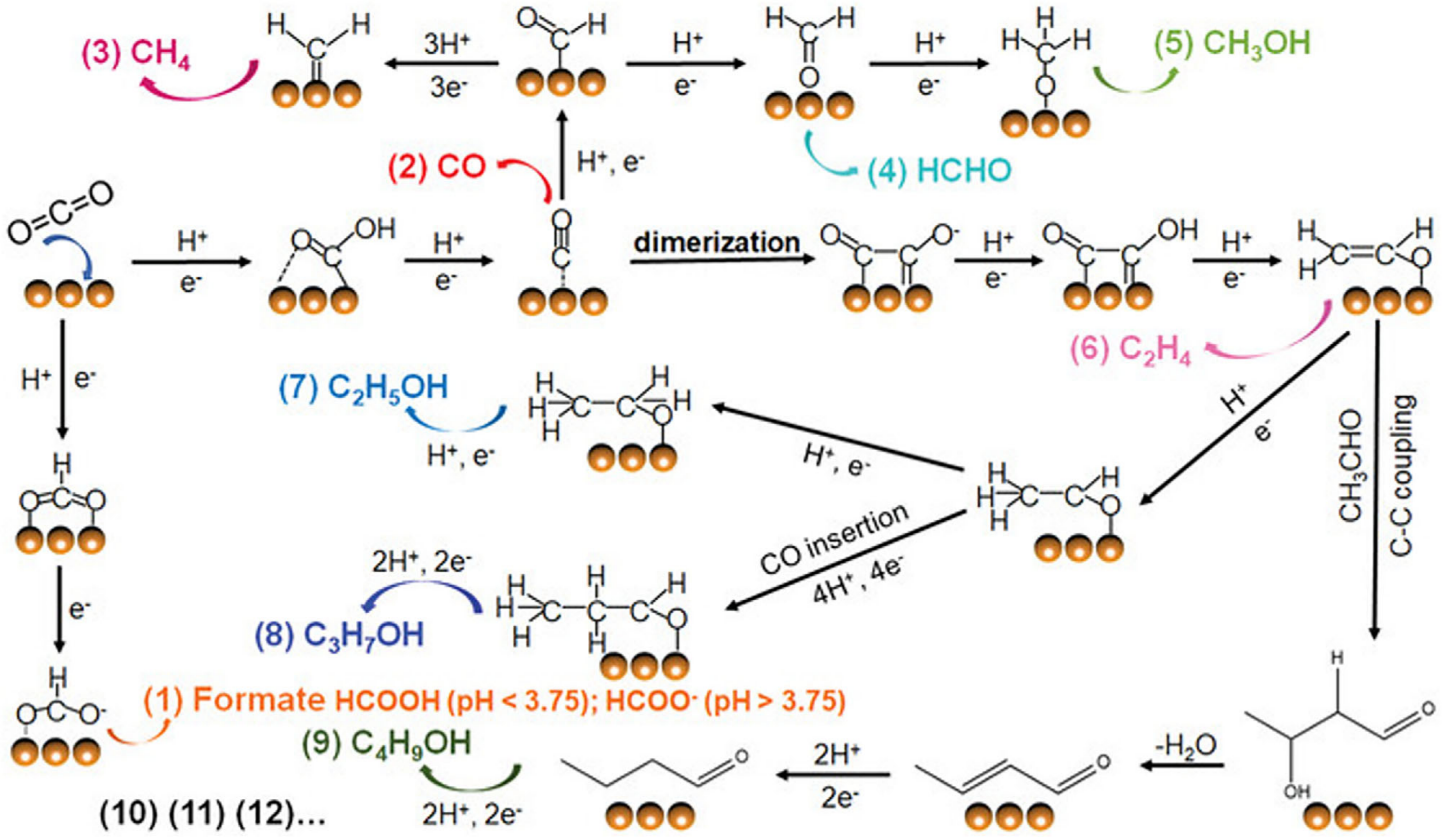
Schematic illustration of the potential pathway for CO_2_RR to C_1_ and C_2+_ products. Reproduced with permission [30]. Copyright 2022, John Wiley and Sons.

The molecular structure of CO_2_ is highly stable, requiring relatively high energies for C═O activation of approximately 800 kJ mol^−1^ [30]. The CO_2_RR involves several steps, including CO_2_ adsorption, CO_2_ radical anion formation, key reaction intermediates via proton‐coupled electron transfer (PCET), and the formation of desired products. To complete these steps, the high energy barriers of each step must be overcome, which necessitates the application of an additional external potential. As shown in Table 1, distinct numbers of multi‐protons and electrons are necessary to generate varying products. As depicted in Figure 2, CO_2_ is adsorbed onto the surface of the catalyst and receives an electron to form the CO_2_ radical anion. Reaction intermediates such as *OCHO or *COOH are generated through the initial PCET process. For *OCHO intermediates, the oxygen atoms of CO_2_ bind to the surface of the catalyst, while the carbon atom is protonated. Through further PCET, either HCOOH or HCOO^–^ is produced, depending on the pH. For *COOH intermediates, the carbon atom of CO_2_ binds to the surface of the catalyst. The subsequent PCET process leads to the formation of *CO intermediates. If the desorption energy of *CO is low, *CO desorption is favored, resulting in the formation of CO products. Conversely, if the *CO intermediates bind strongly to the catalyst surface, the reaction pathway diverges into the C_1_ pathway and the C_2+_ pathway. In the C_1_ pathway, a series of multi‐step PCET processes are carried out on the *CO intermediates, resulting in the formation of various C_1_ products, such as CH_4_, HCHO, and CH_3_OH, depending on the binding strength. In contrast, in the C_2+_ pathway, CO dimerization leads to the formation of C═C coupling, followed by a multi‐step PCET reaction, resulting in C_2+_ products such as C_2_H_4_, C_2_H_5_OH, and C_3_H_7_OH.

### 2.3 In Situ Analysis for CO_2_RR

In situ analysis represents a crucial tool for elucidating the underlying factors behind product formation, quantifying product yields, and formulating precise conclusions regarding reaction sites [31]. This technique is particularly invaluable when dealing with unstable products/species or intricate multistep reaction pathways. In the case of CO_2_RR, comprehending the reaction mechanism involving multiple PCET processes poses a challenge. A fundamental understanding of how the catalyst surface interacts with reactive species during the CO_2_RR remains elusive. Consequently, numerous in situ techniques have been employed to address these knowledge gaps.

**FIGURE 3:**
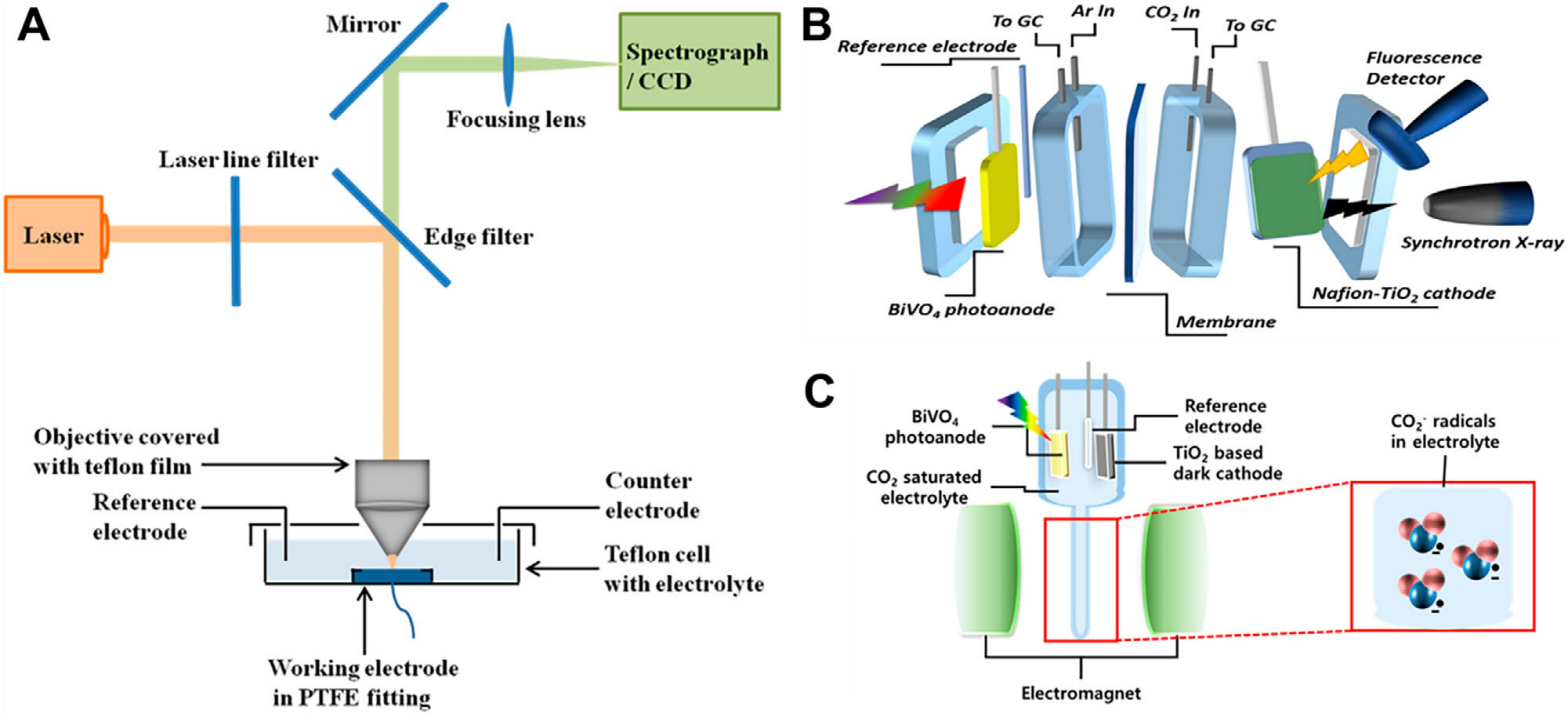
Schematic illustration of specialized cells for in situ analysis corresponding to (A) in situ Raman spectroscopy (B) in situ X‐ray absorption spectroscopy (C) in situ electron paramagnetic resonance spectroscopy. Reproduced with permission [32]. Copyright 2017, American Chemical Society. Reproduced with permission [33]. Copyright 2019, American Chemical Society.

In situ Raman spectroscopy has proven to be a valuable tool for elucidating structural changes in CO_2_, identifying intermediate products present on electrode surfaces, and pinpointing catalytic active sites on catalyst surfaces [32]. As illustrated in Figure 3A, a specialized cell was developed, integrating an in situ Raman spectroscopy setup, to facilitate the investigation of (photo)electrochemical CO_2_RR. This spectroscopy reveals that structural or compositional changes were predominantly observed on various catalyst surfaces during the CO_2_RR.

In situ X‐ray absorption spectroscopy (XAS) is also a useful tool for identifying the compositional and structural dynamics of catalysts during (photo)electrochemical CO_2_RR [33]. XAS analysis is based on the concept of a specific absorption edge, which is further delineated into two distinct regions. The X‐ray absorption near edge structure (XANES) examines the 50 eV vicinity near the absorption edge, while the extended X‐ray absorption fine structure (EXAFS) explores the region extending from a few hundred to approximately 1000 eV beyond the edge. XANES is particularly sensitive to the oxidation state of the X‐ray absorbing atom and its geometric arrangement relative to the surrounding atoms [34, 35]. On the other hand, EXAFS provides insights into the radial distribution of electron density around the absorbing atom, offering valuable information about the bond length and coordination number [36]. As a result, in situ XAS measurements, conducted using a specialized cell as depicted in Figure 3B, enable the observation of changes in the oxidation state and coordination environment of catalysts during CO_2_RR.

In situ electron paramagnetic resonance (EPR) spectroscopy, as illustrated in Figure 3C, is an effective tool for identifying intermediate radicals, such as the CO_2_ radical anion, during CO_2_RR [33]. The utilization of a spin‐trapping agent, such as *N*‐tert‐butyl‐α‐phenylnitrone [33] and α‐phenyl *N*‐tert‐butyl nitrone [37], facilitates the trapping and detection of these intermediate radicals. Once the intermediate radical‐trapping agent complex is constructed, the EPR signal is detected through the unpaired electrons of the intermediate radicals. Therefore, this spectroscopy demonstrates the formation of the intermediate radicals, confirming the occurrence of (photo)electrochemical CO_2_RR at the electrode.

## 3 Interface Engineering of Photocathodes for PEC CO_2_RR

### 3.1 Cocatalyst Engineering

Bare semiconductor surfaces typically lack electrocatalytic activity, making it challenging for them to interact effectively with CO_2_ molecules [29]. Integrating appropriate cocatalysts with semiconductor materials is crucial for enhancing surface catalytic conversion, reducing the overpotential of the reaction, and facilitating the transfer of photoinduced charge carriers to surface reactants [38]. This integration accelerates surface reaction kinetics and improves PEC activity and selectivity. Consequently, the efficacy of this integration is contingent upon meticulous cocatalyst selection, the establishment of robust semiconductor‐cocatalyst interfaces, and the assurance of their uniform distribution across the semiconductor surface [29]. This section is devoted to the categorization of cocatalysts for PEC CO_2_RR into two distinct groups: plasmonic noble metals and non‐noble metals. Plasmonic noble metals are distinguished by their surface plasmon resonance properties, which elucidate their optical effects on PEC CO_2_RR. In contrast, non‐noble metal cocatalysts are primarily characterized by their electrochemical catalytic activity and electron transfer capabilities.

#### 3.1.1 Plasmonic Noble Metal

Plasmonic noble metal cocatalysts incorporate noble metals like Ag and Au, known for their abundance of free‐mobility electrons [39]. Surface plasmon resonance in these cocatalysts originates from the collective oscillations of nanostructures and nanogaps under intense electromagnetic radiation [40, 41, 42]. Such resonance, characterized by incident light interacting with the cocatalyst, enhances the redistribution and conversion of light energy through the re‐emission of plasmon‐induced light, non‐radiative decay to excited carriers (hot electrons and holes), and thermal effects over specific timescales [39].

Regarding PEC reactions, the size effect of plasmonic materials becomes significant, with plasmonic noble metal nanostructures improving light absorption, catalytic activity, selectivity, and efficiency [43, 44, 45, 46, 47]. Hence, plasmonic noble metal cocatalysts, by exciting surface plasmons, could harness broad‐spectrum sunlight, producing high‐energy hot carriers that facilitate PEC CO_2_RR.

**FIGURE 4:**
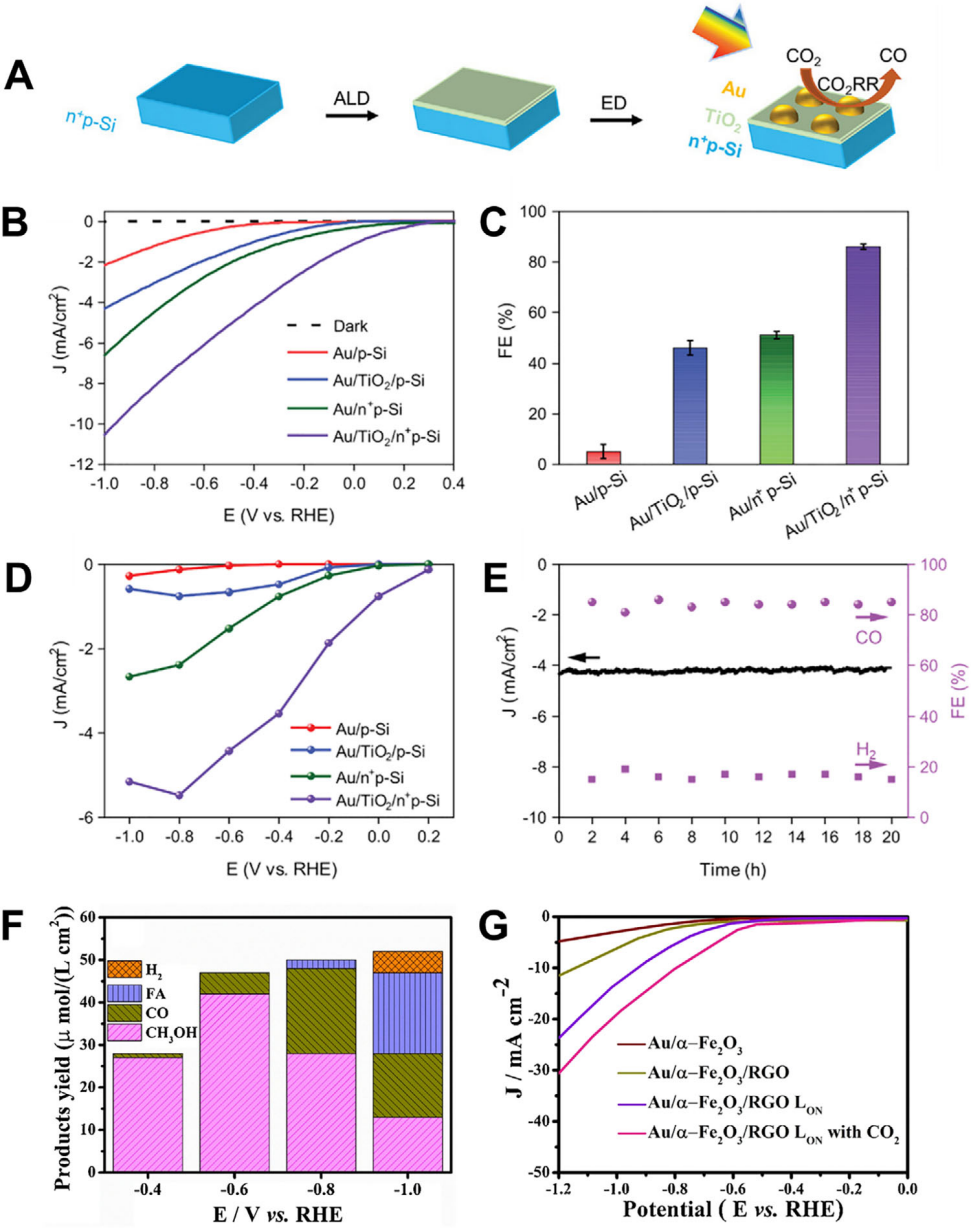
(A) Schematic illustration of the fabrication process of an Au/TiO_2_/n^+^p^–^ Si photocathode. (B) LSV curves, (C) FE_CO_, (D) partial photocurrent density for CO, and (E) long‐term operational stability test of the Au/TiO_2_/n^+^p^–^ Si photocathode. Reproduced with permission [49]. Copyright 2022, John Wiley and Sons. (F) product yield for the Au/α‐Fe_2_O_3_/RGO photocathode at different potentials (V vs. RHE). (G) total photocurrent density of the Au/α‐Fe_2_O_3_/RGO photocathode. Reproduced with permission [51]. Copyright 2021, Elsevier.

For example, Liu et al. designed a CuBi_2_O_4_ inverse opal photocathode modified with plasmonic Ag nanoparticles (Ag NPs) using a sacrificial template method (CuBi_2_O_4_ IOs‐Ag) [48]. The 3D‐ordered structure of CuBi_2_O_4_ inverse opal enabled higher mass transfer rates and light harvesting efficiency. Furthermore, incorporating Ag NPs significantly enhanced the surface charge distribution by forming an ohmic contact with CuBi_2_O_4_. The CuBi_2_O_4_ IOs‐Ag photocathodes showed notable improvements in selectivity for CO production, achieving a faradaic efficiency for CO (FE_CO_) of 92% at 0.2 V vs. reversible hydrogen electrode (RHE), which is 1.6 times greater than that of the pristine CuBi_2_O_4_ thin film. Wang et al. prepared n^+^p^–^ Si coated with a TiO_2_ interlayer and coupled it with plasmonic Au NPs to fabricate photocathodes (Au/TiO_2_/n^+^p^–^ Si) for PEC CO_2_RR to CO [49]. A schematic illustration of the synthesis method is depicted in Figure 4A. Initially, a TiO_2_ layer was deposited on the micro‐pyramid Si surface using an ALD process, after which Au NPs were fabricated on top of the TiO_2_ layer through an electrodeposition method. The Au/TiO_2_/n^+^p^–^ Si photocathodes exhibited an onset potential of +0.24 V vs. RHE, a maximum FE_CO_ of 86%, and a partial photocurrent density for CO of −5.52 mA cm^−2^ at −0.8 V vs. RHE, as shown in Figure 4B–D. Additionally, these photocathodes demonstrated superior long‐term operational stability for CO production under continuous illumination for 20 h, as shown in Figure 4E. Density functional theory (DFT) calculations indicated that the synergistic effect of Au NPs and TiO_2_ enhanced CO_2_ adsorption and expedited the generation of the ^*^COOH intermediate and ^*^CO desorption from active sites. This research group further investigated the localized surface plasmon resonance (LSPR) effect of Au on the TiO_2_ layer, which contributed to increased activity and selectivity for CO production by utilizing hot electrons generated in Au NPs. Bharath et al. designed photocathodes by integrating plasmonic Ag NPs with TiO_2_/RGO (Ag‐TiO_2_/RGO) via a hydrothermal method followed by microwave irradiation [50]. In this composite, Ag NPs not only absorbed visible light but also acted as efficient electron scavengers, thus enhancing PEC performance for CO_2_RR. PEC measurements revealed that the Ag‐TiO_2_/RGO photocathodes achieved a notable total photocurrent density of 23.5 mA cm^−2^ and exhibited low resistance of 125 Ω in a CO_2_‐saturated 1.0 m KOH solution under ultraviolet‐visible (UV–vis) light illumination. Furthermore, the Ag‐TiO_2_/RGO photocathodes displayed a CH_3_OH yield of 85 µmol L^−1^ cm^−2^, a QE of 20%, and a faradaic efficiency for CH_3_OH (FE_CH3OH_) of 60.5% at an onset potential of −0.7 V vs. Ag/AgCl. Bharath et al. also fabricated photocathodes featuring plasmonic‐Au and RGO‐incorporated α‐Fe_2_O_3_ nanorods (Au/α‐Fe_2_O_3_/RGO) aiming for highly selective CH_3_OH production [51]. The synergistic effects among the size‐dependent properties of α‐Fe_2_O_3_, the plasmonic nature of Au, and the chemical interactions of Au, RGO, and α‐Fe_2_O_3_ nanorods resulted in a higher band gap for Au/α‐Fe_2_O_3_/RGO (2.60 eV). This band gap allowed the composite to absorb more intensely in the high‐energy range of the visible spectrum, enabling the efficient use of photogenerated electrons and reducing e^–^/h^+^ pair recombination effects. Consequently, the Au/α‐Fe_2_O_3_/RGO photocathodes exhibited an impressive photocurrent density of −31.5 mA cm^−2^ and achieved a maximum CH_3_OH yield of 43 µmol L^−1^ cm^−2^, as shown in Figure 4F,G. Additionally, the photocathodes attained QE and FE_CH3OH_ of 21.5% and 91%, respectively, at −0.6 V vs. SCE in a CO_2_‐saturated 0.1 m KOH electrolyte under illumination.

Li et al. developed a photocathode for PEC CO_2_RR by dispersing Ag NPs onto a Cu‐modified mesoporous TS‐1 zeolite (Ag/Cu‐TS‐1) [52]. The fabrication process involved an ion exchange method followed by an in situ photodeposition method, resulting in the synthesis of Cu‐TS‐1 via ion exchange and the highly dispersed Ag NPs onto Cu‐TS‐1 through in situ photodeposition. The Ag/Cu‐TS‐1 photocathode showed exceptional light absorption and efficient separation of e^–^/h^+^ pairs, enhancing CO_2_RR. This improvement was attributable to the heterostructure of Cu_2_O/CuO and the LSPR effect of the Ag NPs. In PEC performance terms, the Ag/Cu‐TS‐1 photocathode demonstrated conversion of CO_2_ into CH_3_OH and C_2_H_5_OH at rates of 5.64 and 2.62 µmol cm^−2^ h^−1^, respectively, at −0.6 V vs. RHE in a CO_2_‐saturated 0.1 m KHCO_3_ electrolyte. Zhang et al. constructed a plasmonic Ag‐adorned Cu_2_O nanowire (Cu_2_O/Ag) photocathode for PEC CO_2_RR targeting C_2+_ products [53]. The LSPR effect of Ag contributed to both enhanced rapid separation of e^–^/h^+^ pairs and improved surface catalytic reactions for C_2+_ product generation. To substantiate the LSPR effect in the Cu_2_O/Ag photocathode, the UV–vis absorption spectrum was used to confirm increased light absorption due to the presence of Ag NPs. Additional in situ attenuated total reflection infrared spectroscopy (ATR‐IR) results indicated that incorporating Ag NPs improved the formation and adsorption of the CH_3_O^*^ intermediate. PEC tests showed the Cu_2_O/Ag photocathode achieved a faradaic efficiency for CH_3_COOH (FE_CH3COOH_) of 47.7%, with a generation rate of 212.7 µmol cm^−2^ h^−1^ at −0.7 V vs. RHE under illumination.

#### 3.1.2 Non‐Noble Metal

Due to the high structural stability of CO_2_ molecules, many semiconductors face challenges in selectively facilitating CO_2_RR. In such scenarios, cocatalysts play a crucial role in minimizing the overpotential required for CO_2_RR and enhancing the kinetics to improve overall selectivity. The electrical conductivity of the cocatalyst, along with its adsorption and desorption capabilities based on binding energies with relevant reaction species—especially those with moderate binding affinities for key intermediates—can lead to higher intrinsic CO_2_RR activity [54, 55, 56]. This, in turn, facilitates the promotion of CO_2_RR at lower overpotentials, thereby enhancing onset potentials.

Noble metals, such as Ag and Au, remain efficient catalysts for converting PEC energy into CO_2_. However, the use of noble metal‐based catalysts faces significant limitations due to their high cost and limited availability, which constrain commercial implementation [38, 57, 58]. Consequently, extensive research efforts have been directed toward developing alternative catalysts for CO_2_RR based on non‐noble metal materials, including transition metals. These materials aim to provide low‐cost, high‐activity, and long‐term stability for CO_2_RR, addressing the challenges associated with noble metal cocatalysts [38, 59–61].

**FIGURE 5:**
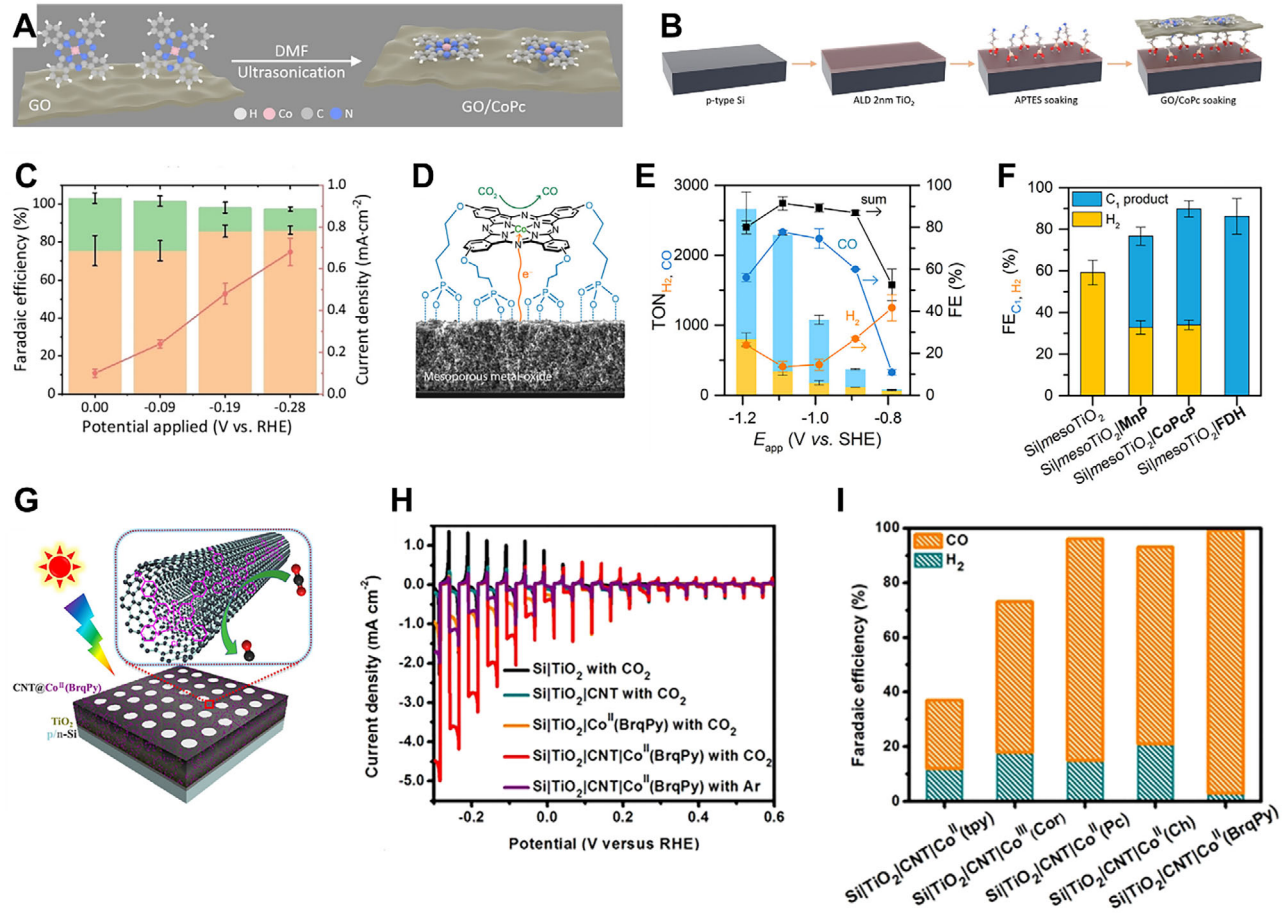
(A) Schematic illustration of the preparation of GO/CoPC and (B) STA‐GO/CoPc photocathode. (C) photocurrent density with FE for H_2_ and CO of the STA‐GO/CoPc photocathode. Reproduced with permission.[62]. Copyright 2022, John Wiley and Sons. (D) Schematic representation of the molecular structure of mesoTiO_2_|CoPcP. (E) TON for CO and H_2_ of Ti|mesoTiO_2_|CoPcP. (F) FE for C_1_ and H_2_ products yielded by Si|mesoTiO_2_|CoPcP and other counterpart photocathodes. Reproduced with permission [63]. Copyright 2021, American Chemical Society. (G) Illustration of the Si|TiO_2_|CNT|Co^II^ (BrqPy) photocathode. (H) LSVs and (I) FE for H_2_ and CO of the Si|TiO_2_|CNT|Co^II^ (BrqPy) photocathode compared with other groups. Reproduced with permission [64]. Copyright 2022, John Wiley and Sons.

**FIGURE 6:**
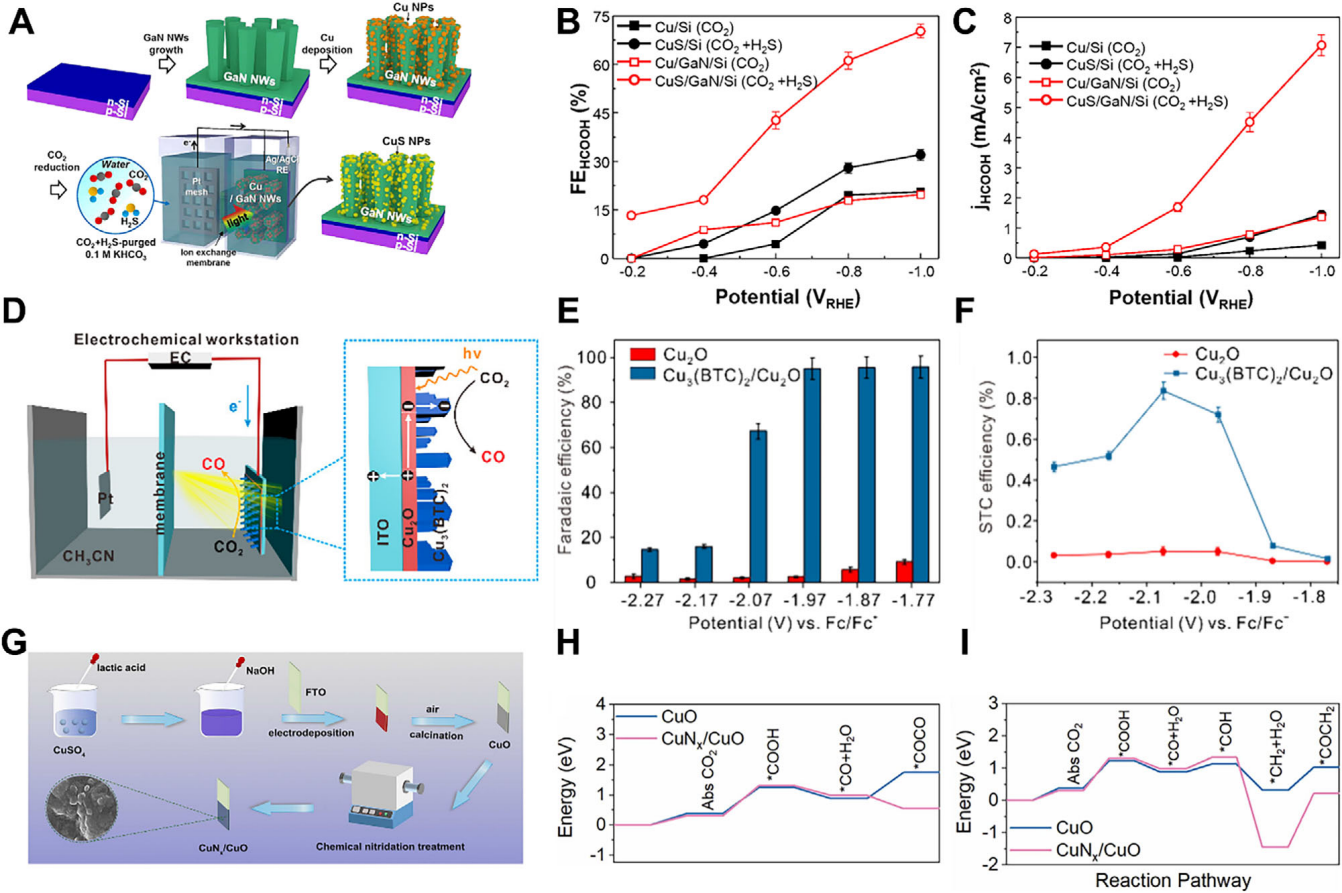
(A) Schematic illustration of the CuS/GaN/Si photocathode synthesis and PEC experiments. (B) FE_HCOOH_ and (C) *j*_COOH_ of the CuS/GaN/Si photocathode. Reproduced with permission [66]. Copyright 2021, American Chemical Society. (D) Schematic depiction of the PEC experiments of Cu_3_(BTC)_2_/Cu_2_O photocathode. (E) FE_CO_ and (F) STC efficiency of the Cu_2_O and Cu_3_(BTC)_2_/Cu_2_O/ITO photocathode. Reproduced with permission [69]. Copyright 2019, American Chemical Society. (G) Schematic illustration of the CuN*_x_*/CuO photocathode synthesis. The calculated energy barrier for forming (H) EtOH and (I) acetate for the CuO and CuN*_x_*/CuO photocathode. Reproduced with permission [70]. Copyright 2022, Elsevier.

Among these, cobalt molecular catalysts are widely used as cocatalysts for the photocathode in PEC CO_2_RR. For instance, Shang et al. prepared a p‐type silicon photocathode incorporating a cobalt phthalocyanine molecular catalyst immobilized on graphene oxide (GO/CoPc) [62]. Initially, CoPc molecules were immobilized on GO through ultrasonication in a DMF solution, taking advantage of GO's conductive properties and capability to facilitate electron transfer, as illustrated in Figure 5A. Subsequently, p‐Si wafers, coated with a protective TiO_2_ layer (Si‐TiO_2_), were treated with a solution of (3‐aminopropyl)triethoxysilane (APTES), as depicted in Figure 5B. Following this treatment, the Si‐TiO_2_‐APTES (STA) substrate was immersed in a GO/CoPc aqueous dispersion, forming a monolayer‐like coating on the surface through electrostatic and hydrogen bonding interactions between the amine groups of APTES and the carboxylic acid groups of GO. The STA‐GO/CoPc achieved a photocurrent density of 0.7 mA cm^−2^, while simultaneously attaining a maximum FE_CO_ of 86% at −0.28 V vs. RHE, as observed in Figure 5C. Moreover, STA‐GO/CoPc exhibited a remarkably low onset potential of −0.36 V vs. RHE, displaying a FE_CH3OH_ of 8% at −0.62 V vs. RHE. As shown in Figure 5D, Roy et al. constructed a hybrid photocathode structure comprising a cobalt phthalocyanine catalyst with four phosphonic acid anchoring groups (CoPcP) immobilized on mesoTiO_2_, which coated a p‐Si photocathode (Si|mesoTiO_2_|CoPcP) [63]. Incorporating the four phosphonic acid anchoring groups facilitated the immobilization of the CoPcP catalyst on meso TiO_2_. To assess the impact of the CoPcP catalyst on mesoTiO_2_, they synthesized a mesoTiO_2_|CoPcP hybrid electrode. As depicted in Figure 5E, the resulting mesoTiO_2_|CoPcP photocathode demonstrated highly selective CO_2_ to CO conversion, achieving a turnover number for CO (TON_CO_) of 1949 ± 5 after 2 h of controlled‐potential electrolysis at a 550 mV overpotential in a 0.5 m KHCO_3_ aqueous electrolyte. In addition, when combined with a p‐Si photocathode, the Si|mesoTiO_2_|CoPcP exhibited a TON_CO_ of 939 ± 132 with 66% CO selectivity under 0.5 m KHCO_3_ conditions, as observed in Figure 5F. Wen et al. prepared Co^II^ (BrqPy) (BrqPy = 4′,4′′‐bis(4‐bromophenyl)‐2,2′:6′,2′′:6′′,2′′′‐quaterpyridine) molecular catalysts with multiwalled carbon nanotubes (CNT) on TiO_2_‐protected p/n‐Si photocathodes (Si|TiO_2_|CNT|Co^II^ (BrqPy)) [64], as illustrated in Figure 5G. First, a p/n‐Si wafer was covered with TiO_2_ through atomic‐layer deposition (ALD). Then, a CNT layer was drop‐cast onto the Si|TiO_2_ layer. Finally, a DMF solution containing Co^II^ (BrqPy) molecular catalysts was drop‐cast, and the catalysts were immobilized on the CNT layer through π─π stacking interactions. The hybrid photocathodes, benefiting from the highly conductive nature of CNT, achieved a remarkable photocurrent density of up to −1.4 mA cm^−2^ at −0.11 V vs. RHE, as seen in Figure 5H. Furthermore, due to the exceptional activity and selectivity of the cobalt molecular catalyst for CO_2_RR, the total FE, and FE_CO_ reached 99% and 97%, respectively, as revealed in Figure 5I. These results surpassed other hybrid photocathodes employing more complex configurations. Leung et al. employed cobalt(II) bis(terpyridine) molecular catalysts (CotpyP) as cocatalysts to construct a highly efficient photocathode for CO_2_RR [65]. This hybrid photocathode consisted of a p‐type silicon photocathode coated with a mesoporous TiO_2_ layer with anchored CotpyP catalysts (Si|mesoTiO_2_|CotpyP). The mesoporous TiO_2_ layer protected the Si, allowing for high loading of CotpyP catalysts and ample surface contact with the electrolyte due to its high surface area. Photoelectrochemical tests indicated CO and HCOO^–^ production in aqueous acetonitrile (MeCN) with 0.1 m tetrabutylammonium tetrafluoroborate (TBABF_4_) and pure CO_2_‐saturated 0.1 m KHCO_3_. The turnover number for CO_2_RR reached 381 during a 24 h test in aqueous MeCN. Furthermore, nanowires (NWs), NPs, metal oxides, and MOFs, which consist of non‐noble metals, are also widely used as photocathodes in PEC CO_2_RR. For example, Dong et al. designed a photocathode comprising CuS‐covered GaN NWs on silicon wafers (CuS/GaN/Si) to convert H2S‐containing CO_2_ mixture gas to HCOOH efficiently [66]. The fabrication involved thermally evaporating Cu NPs and plasma‐assisted molecular beam epitaxy (MBE) of GaN NWs on Si wafers. PEC experiments were conducted in a 0.1 m KHCO_3_ solution purged with CO_2_ and H_2_S mixture gas under 1‐sun illumination, as shown in Figure 6A. Notably, Cu NPs spontaneously transformed into CuS NPs during the PEC experiment. Compared to other photocathodes of Cu/Si, CuS/Si, and Cu/GaN/Si, the CuS/GaN/Si photocathode revealed an outperforming faradaic efficiency for HCOOH (FE_HCOOH_) of 70.2% at −1.0 V vs. RHE and achieved a maximum current density for HCOOH of 7.07 mA cm^−2^, as shown in Figure 6B,C. Dong et al. fabricated Bi NP cocatalysts supported on GaN NWs photocathodes for PEC CO_2_RR to HCOOH [67]. Focusing on the electronic interaction between Bi NPs and GaN NWs grown on a planar Si wafer (Bi/GaN/Si), the strong electronic interaction enhanced CO_2_ conversion due to electron sharing between Bi NPs and GaN NWs. The Bi/GaN/Si photocathode demonstrated outstanding selectivity for HCOOH, achieving a FE_HCOOH_ of ∼98% at −0.3 V vs. RHE, a high current density of 10.3 mA cm^−2^ at −0.6 V vs. RHE, and sustained stable operation for 12 h under 1‐sun illumination. This underlines the critical role of electronic interactions between photocathodes and cocatalysts in enhancing PEC performance. Zhou et al. showcased a novel nanoarchitecture with a Sn NP/GaN NW/Si photocathode for aqueous PEC reduction of CO_2_ to HCOOH [68]. Integrating defect‐free GaN NWs grown on a planar Si via MBE with electrodeposited Sn NPs, the photocathode's PEC testing revealed exceptional performance, unprecedented TOF of 107 min^−1^, a total current density of 17.5 mA cm^−2^, and a high FE_HCOOH_ of 76.9% at a low potential of −0.53 V vs. RHE under 1‐sun illumination, corresponding to a productivity of 201 µmol cm^−2^ h^−1^. The photocathode also exhibited a high TON for HCOOH production, reaching 64,000 during stable operation over 10 h. DFT calculations suggested that the synergistic effects of covalent Ga─C bonding and ionic‐like Sn‐O bonding played a crucial role in activating CO_2_, contributing to the remarkable activity and selectivity for CO_2_RR. As presented in Figure 6D, Deng et al. developed a Cu_2_O photocathode coated with MOFs named Cu_3_(BTC)_2_ (BTC = benzene‐1,3,5‐tricarboxylate) for PEC CO_2_RR to CO [69]. The Cu_3_(BTC)_2_ coating played multiple roles in the PEC CO_2_RR process, including preventing photocorrosion of the Cu_2_O layer, facilitating electron transfer, and providing catalytic active sites for CO_2_RR. PEC performance was measured in a CO_2_‐saturated MeCN containing 0.1 m tetrabutylammonium hexafluorophosphate (TBAPF_6_). Results indicated that the Cu_3_(BTC)_2_/Cu_2_O/ITO photocathode reached a maximum FE_CO_ of about 95% at applied potentials ranging from −1.77 to −1.97 V vs. ferrocene/ferrocenium (Fc/Fc^+^), as displayed in Figure 6E. Furthermore, the solar‐to‐CO (STC) efficiency of this photocathode reached 0.83% at −2.07 V vs. Fc/Fc^+^ under AM 1.5G illumination, as observed in Figure 6F. Long‐term chronoamperometry indicated that the photocathode maintained a nearly constant current density under visible light and a steady photocurrent density under chopped visible light, highlighting its stability and performance under varying light conditions.

As illustrated in Figure 6G, Wang et al. constructed a hybrid photocathode structure comprising CuO adorned with asymmetric Cu─N sites (CuN*_x_*/CuO) for PEC CO_2_RR [70]. The CuN*_x_*/CuO photocathode exhibited a faradaic efficiency towards C_2_ products (FE_C2_) of 15.2%, accompanied by a photocurrent density of −1.0 mA cm^−2^ at 0.2 V vs. RHE in CO_2_‐saturated 0.1 m KHCO_3_ under AM 1.5G simulated sunlight. DFT calculations demonstrated that the adsorption of OCCO^*^ and ^*^COCH_2_ intermediates on Cu─N sites, crucial for the formation of C_2_ products, was more favorable than on Cu─Cu sites, as presented in Figure 6H,I. Theoretical calculations also indicated that the CuN*_x_*/CuO photocathode had higher electron migration efficiency than CuO due to the asymmetric d‐p orbitals at Cu─N sites, which lowered the energy barrier for C─C coupling. Roh et al. fabricated Si NWs connected with a Cu NP ensemble to create photocathodes (Cu NPs/Si NWs) for PEC CO_2_RR to C_2_H_4_ [71]. Assessed under 100 mW cm^−2^ air mass (AM) 1.5 simulated sunlight in CO_2_‐purged 0.1 m KHCO_3_, the photocathode achieved a selectivity for CO_2_RR to C_2_H_4_ with a faradaic efficiency (FE_C2H4_) of approximately 25% and demonstrated activity with partial current densities exceeding 2.5 mA cm^−2^ at −0.50 V vs. RHE. Moreover, the Cu NPs/Si NWs photocathode exhibited long‐term stability, maintaining PEC CO_2_RR under 50 h of continuous bias and illumination. Kim et al. constructed a metal‐insulator‐semiconductor (MIS) structure comprising Cu/TiO_2_/p‐Si photocathodes for PEC CO_2_RR to multicarbon products [72]. They also investigated the effects of ionomer bilayer coatings, specifically Nafion atop Sustainion, on the Cu surface of the Cu/TiO_2_/p‐Si MIS photocathodes. PEC testing indicated that Cu/TiO_2_/p‐Si photocathodes coated with Nafion on top of Sustainion revealed partial current densities for C_2_H_4_ ranging from −0.9 mA cm^−2^ to −2.3 mA cm^−2^ in CO_2_‐saturated 0.1 m CsHCO_3_ under wet‐side illumination, compared to the condition without ionomer bilayers on the Cu surface. This underscores the enhancement of both activity and selectivity for C_2_H_4_ due to the Cu cocatalyst and bilayer coatings.

### 3.2 Junction Engineering

A heterojunction is typically defined as a structure composed of two or more different semiconductors with a contacting interface, distinct band energy levels, a matching crystal lattice, and similar thermal expansion coefficients [73]. Heterojunctions can be classified into type‐I, type‐II (including p–n junctions), and Schottky barrier junctions (metal–semiconductor). Semiconductor heterojunctions often involve interfacing two semiconductor materials with differing Fermi‐level energies, creating a built‐in electric field that promotes the separation of electrons and holes upon light excitation.

In the case of type‐I heterojunctions, two semiconductors with overlapping band structures typically exhibit one semiconductor with a more negative CB position and a more positive VB position than the other semiconductor. Conversely, in type‐II heterojunctions, the two semiconductors have staggered band structures where electrons transition from a more negative CB to a less negative CB, and holes move in the opposite direction. In type‐II heterojunctions, photogenerated electrons and holes are efficiently separated, allowing for e^–^/h^+^ pairs to be excited using a greater number of photons [73].

Schottky barrier junctions create band bending near the semiconductor‐metal catalyst interface, facilitating electron transfer from the semiconductor to the metal. Nevertheless, this section focused solely on semiconductor heterojunctions, as Schottky barrier junctions overlap with concepts such as cocatalysts, and there is extensive prior research in this domain.

**FIGURE 7:**
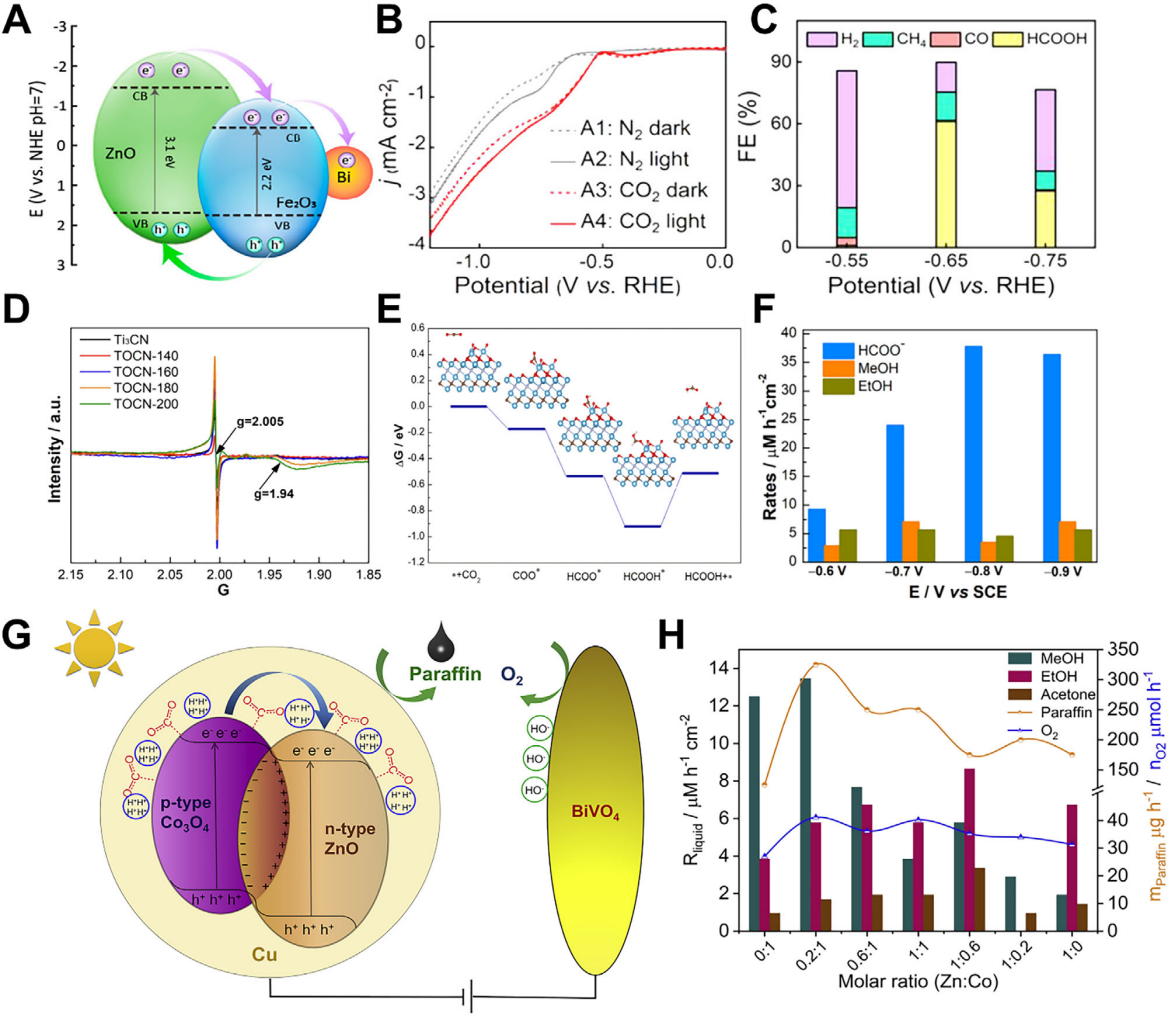
(A) Proposed PEC CO_2_RR mechanism of 1D Bi@ZFO NTs photocathode. (B) LSV curves and (C) FE of H_2_, CH_4_, CO, and HCOOH of the 1D Bi@ZFO NTs photocathode. Reproduced with permission [74]. Copyright 2022, American Chemical Society. (D) EPR spectra and (E) calculated Gibbs free energy of forming HCOOH. (F) Various product yield rates of the TiO_2_/Ti_3_CN MXene photocathode. Reproduced with permission [79]. Copyright 2023, Elsevier. (G) Proposed mechanism of PEC CO_2_RR to paraffin products and (H) paraffin yield rate of Zn*_x_*:Co*_y_* @Cu photocathode. Reproduced with permission [84]. Copyright 2020, Elsevier.

For example, Quyang et al. prepared a photocathode tailored for PEC CO_2_RR to HCOOH, consisting of Bi‐modified 1D ZnO/α‐Fe_2_O_3_ nanotubes (1D Bi@ZFO NTs) [74]. The formation of an n‐n heterojunction between the narrow bandgap of α‐Fe_2_O_3_ and the wide bandgap of ZnO was instrumental in enhancing charge transfer, establishing an internal electric field conducive to driving the transfer of photoexcited charges, as illustrated in Figure 7A. Additionally, depositing Bi onto the ZnO/α‐Fe_2_O_3_ heterojunction potentially increased the carrier concentration at the electrode surface, thereby enhancing the efficiency of photogenerated charge separation. Consequently, the Bi@ZFO NTs photocathode indicated a low onset potential of −0.53 V vs. RHE, a low Tafel slope of 101.2 mV dec^−1^, and achieved a high FE_HCOOH_ of 61.2% at −0.65 V vs. RHE, maintaining stability over 4 h under visible light, as shown in Figure 7B,C. Jiang et al. reported a photocathode incorporating CuO onto graphitic carbon nitride (g‐C_3_N_4_) supported on carbon paper (CuO/g‐C_3_N_4_/carbon paper) for PEC CO_2_RR to CH_3_OH [75]. Establishing a type‐I heterojunction facilitated photogenerated electron transfer from the CB of g‐C_3_N_4_ to the CB of CuO, which has a relatively less negative CB energy level under illumination. Concurrently, favorable hole transfer occurred from the VB of g‐C_3_N_4_ to the VB of CuO, which held a lower VB energy. As a result, the CuO/g‐C_3_N_4_/carbon paper photocathode exhibited a high IPCE, an increased photocurrent response, and a remarkable FE of 75% and QE of 8.9%, respectively, for CH_3_OH production. Pan et al. presented a photocathode consisting of a Cu catalyst decorated with flower‐like CeO_2_ NPs and CuO NPs, functioning as n‐type and p‐type semiconductors, respectively [76]. The formation of a p‐n heterojunction with CeO_2_ NPs/CuO NPs facilitated the synergistic migration of photoexcited electrons and holes, leading to exceptional PEC performance for CO_2_RR. Thus, the CeO_2_ NPs/CuO NPs/Cu photocathode revealed a CH_3_OH yield rate of 3.44 µmol cm^−2^ h^−1^ and exhibited a high FE_CH3OH_ of approximately 60% at – 1.0 V vs. saturated calomel electrode (SCE) under visible light irradiation. Zheng et al. utilized zinc phthalocyanine (ZnPc) integrated with carbon nitride nanosheets as a photocathode for PEC CO_2_RR to CH_3_OH [77]. The aligned energy bands between ZnPc and carbon nitride facilitated electron transfer and reduced recombination of the e^–^/h^+^ pair. Simultaneous exposure to light and an external voltage ensured that reductive electrons were generated not only through light excitation but also by the external voltage. This augmented the transfer rate of photogenerated electrons from the CB of carbon nitride to the LUMO of ZnPc. Consequently, the ZnPc/carbon nitride photocathode exhibited a predominant CH_3_OH product with a yield of 13 µmol L^−1^ after 8 h at −1.0 V vs. SCE. Tarek et al. developed a heterostructured CdS─CuFe_2_O_4_ photocathode to convert CO_2_ into CH_3_OH [78]. The CdS─CuFe_2_O_4_ photocathode exhibited a higher IPCE of 12.09% compared to CuFe_2_O_4_ with an IPCE of 7.28% at 470 nm, illustrating effective visible light absorption during PEC CO_2_RR. Within the CdS─CuFe_2_O_4_ heterojunction, the CB of CdS served as the site for CO_2_RR, capturing photogenerated electrons originating from CuFe_2_O_4_, while water oxidation occurred at the VB of CuFe_2_O_4_. Accordingly, PEC performance indicated that the CdS─CuFe_2_O_4_ photocathode revealed an FE_CH3OH_ of 72% and a QE_CH3OH_ of 16.9% and recorded a maximum CH_3_OH yield of 23.8 µmol L^−1^ cm^−2^ in CO_2_‐saturated 0.1 m NaHCO_3_ electrolyte. Xu et al. prepared a 2D heterojunction of TiO_2_/Ti_3_CN MXene as a photocathode for PEC CO_2_RR, synthesized using a simple hydrothermal oxidation method [79]. The 2D TiO_2_/Ti_3_CN heterojunction, with its large specific surface area, exceptional light absorption ability, and abundant Ti^3+^ species, facilitated the efficient generation and migration of e^–^/h^+^ pairs. To confirm the impact of Ti^3+^, electron paramagnetic resonance (EPR) spectra were obtained, as depicted in Figure 7D. The EPR spectra demonstrated widespread detection of Ti^3+^ species, proving beneficial for trapping charge carriers and mitigating the recombination of e^–^/h^+^ pairs. Furthermore, DFT calculations suggested that the 2D TiO_2_/Ti_3_CN heterojunction could spontaneously adsorb CO_2_ molecules and stabilize key intermediates crucial for HCOOH production, as shown in Figure 7E. Consequently, the novel PEC system, composed of Pd@TiO_2_/Ti_3_CN||SCE||BiVO_4_, effectively produced HCOO^–^, CH_3_OH, and C_2_H_5_OH with a remarkable formation rate of 45.6 µM cm^−2^ h^−1^, as depicted in Figure 7F. Lu et al. developed a photocathode for PEC CO_2_RR to C_2_H_5_OH, comprising arrays of 0D/1D CuFeO_2_/CuO nanowire heterojunction arrays synthesized through an in situ method [80]. Due to the comparable energy band gaps of CuO and CuFeO_2_, the photogenerated electrons originating from the CB of CuFeO_2_ migrated to the surface of CuO, while photogenerated holes derived from the VB of CuO moved to CuFeO_2_. This arrangement suppressed the recombination of e^–^/h^+^ pairs under the built‐in electric field of the heterojunction. The PEC performance of the CuFeO_2_/CuO nanowire photocathode exhibited an impressive faradaic efficiency for C_2_H_5_OH (FE_C2H5OH_) of 66.73% at −0.6 V vs. Ag/AgCl. Zhang et al. designed a CuFeO_2_/TNNTs photocathode, incorporating high‐temperature‐durable n‐type Nb‐doped TiO_2_ nanotube arrays (TNNTs) and p‐type CuFeO_2_ for PEC CO_2_RR [81]. Initially, TNNTs were synthesized through anodic oxidation on TiNb alloy sheets. Subsequently, CuFeO_2_/TNNTs were constructed by coating precursor solution on TNNTs, followed by annealing in an argon atmosphere. The high heat stability of TNNTs preserved the well‐maintained structure of regular nanotube arrays. Additionally, TNNTs exhibited semiconductor properties comparable to those of n‐type TiO_2_, enabling their integration with p‐type CuFeO_2_ to form a p‐n heterojunction. As a result, the CuFeO_2_/TNNTs photocathode exhibited high light absorption and accelerated carrier transport due to a suitable band gap and the presence of the p‐n heterojunction. In addition, the CuFeO_2_/TNNTs photocathode demonstrated an outstanding photocurrent of 80 µA cm^−2^, resulting in C_2_H_5_OH production at a rate of 3.3 µmol/5h·cm^−2^. Wang et al. presented a photocathode, designated as BCW‐X, which was achieved by depositing a Bi_2_WO_6_/BiOCl heterojunction onto an F‐SnO_2_ substrate through an in situ hydrothermal process [82]. Notably, the exposed pristine (101) crystal plane of BiOCl transformed into the (112) plane in the heterojunction, facilitated by the excellent compatibility between the (112) planes of BiOCl and the (113) planes of Bi_2_WO_6_. Simultaneously, the heterojunctions in BCW‐X maintained a 2D layered structure, thereby enhancing the efficiency of e^–^/h^+^ pair separation. PEC experiments were conducted in a BCW‐6|KHCO_3_|BiVO_4_ PEC cell under illumination from an Xe lamp, with an external voltage ranging from −0.6 to 1.1 V. The BCW‐6|KHCO_3_|BiVO_4_ system achieved a C_2_H_5_OH yield rate of 600 µmol h^−1^ g^−1^ with an exceptional selectivity of 80% at −1.0 V.

Wang et al. reported, for the first time, a g‐C_3_N_4_/ZnTe type‐II heterojunction photocathode for PEC CO_2_RR to C_2_H_5_OH [83]. This heterojunction facilitated the efficient separation of photoexcited e^–^/h^+^ pairs and promoted electron transfer from ZnTe to g‐C_3_N_4_, driven by the establishment of an interfacial internal electric field (IEF) created between the two semiconductors. The g‐C_3_N_4_/ZnTe photocathode displayed a remarkable C_2_H_5_OH production rate of 17.1 µmol cm^−2^ h^−1^ at −1.1 V vs. Ag/AgCl. Furthermore, DFT calculations suggested that the collaboration between ZnTe, with high CO_2_ adsorption ability, and g‐C_3_N_4_, rich in pyridinic N, played a role as a CO‐producing site, achieving the C─C coupling process through the adsorption of CO with proton‐coupled electron transfer. Wang et al. utilized 3D C/N‐doped heterojunctions of Zn*_x_*:Co*_y_* @Cu as a photocathode for PEC CO_2_RR to paraffin products [84]. The Zn*_x_*:Co*_y_* @Cu photocathode consisted of p‐type semiconductor Co_3_O_4_ and n‐type semiconductor ZnO on Cu foam, forming heterojunctions with various active sites that led to outstanding C─C coupling for paraffin product generation. Upon exposure to light irradiation in a PEC cell, the Zn*_x_*:Co*_y_* @Cu photocathode generated photoexcited e^–^/h^+^ pairs, which were rapidly separated by the built‐in electric field, leading to enhanced mobilities of charge carriers, as illustrated in Figure 7G. Electrons could migrate from the CB of p‐type Co_3_O_4_ to the CB of n‐type ZnO, while holes were either trapped by electrons from the PEC cell circuit or reacted with OH. Therefore, the high concentration of photoelectrons was captured by protons on the surface, resulting in the formation of abundant active hydrogen atoms capable of converting multiple CO_2_ molecules into paraffin products. The Zn*_x_*:Co*_y_* @Cu photocathode demonstrated its optimal PEC performance by achieving a paraffin yield rate of 325 µg h^−1^ at −0.4 V vs. SCE, all while avoiding the release of H_2_, as observed in Figure 7H.

### 3.3 Nanostructure Engineering

Nanostructure engineering is an effective method for enhancing the performance of photocathodes by manipulating the dimensions and morphology of photocathode materials at the nanometer scale [85, 86]. Notable examples of these nanostructures include nanorods, nanowires, dendritic structures, core/shell structures, as well as highly nanoporous and hollow structures. The benefits of employing such nanostructures are manifold. They facilitate enhanced light absorption through scattering and reduce bulk recombination. Additionally, these structures increase the specific surface area, leading to a corresponding increase in active sites [87].

Furthermore, nanostructure engineering involves tuning the crystallographic orientations. Semiconductor nanocrystals typically exhibit anisotropic characteristics, composed of face‐dependent electronic structures, adsorption energy/reactive sites, and photocorrosion resistance, attributable to different atomic configurations and coordination on various crystal facets [88, 89, 90, 91, 92, 93]. Overall, synthesizing nanostructures with a high aspect ratio improves light harvesting and charge separation in the semiconductor bulk [55]. Additionally, nanostructures that expose selective facets, favorable for CO_2_RR kinetics, enhance charge separation at the surface.

**FIGURE 8:**
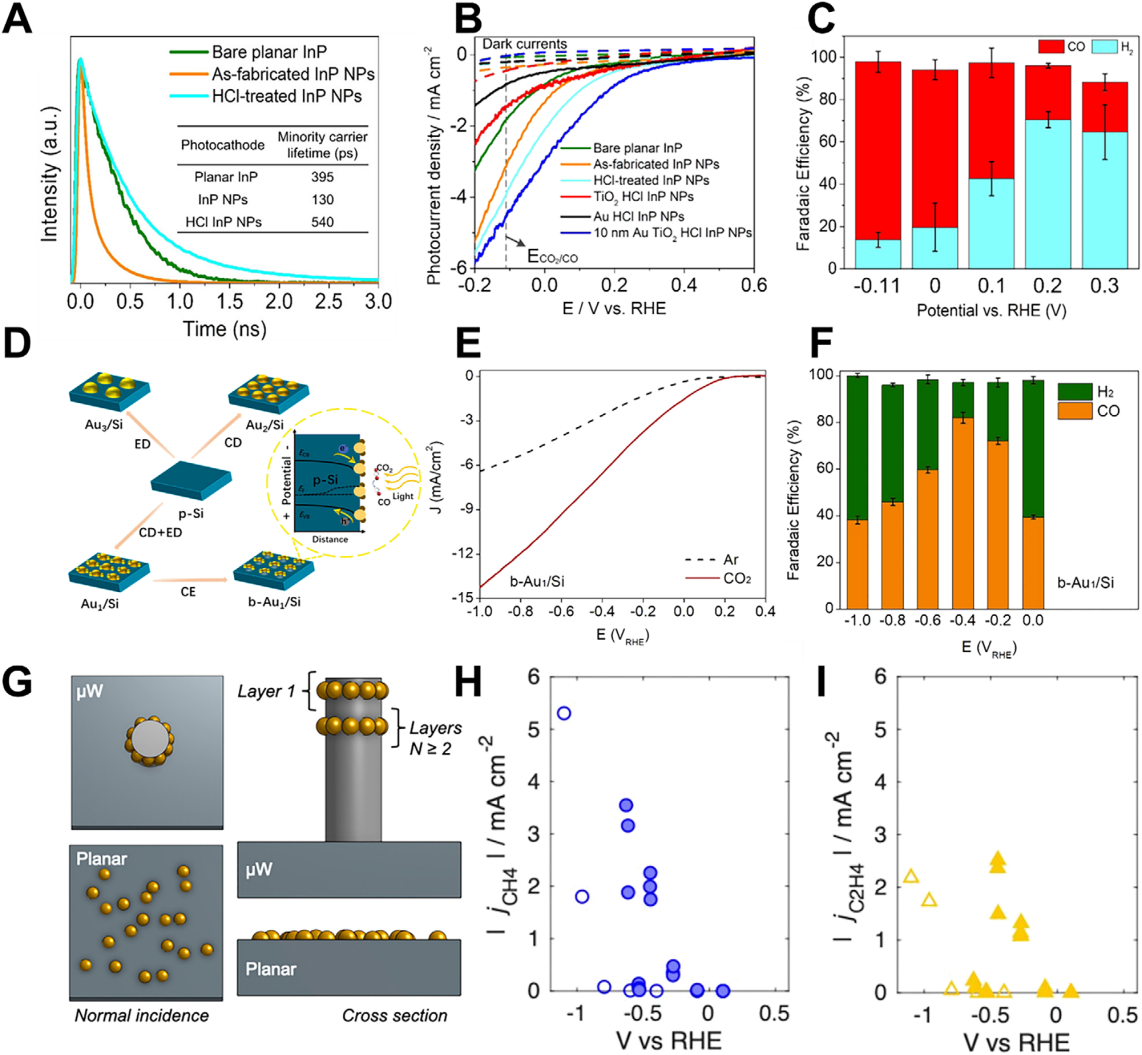
(A) TRPL spectra of samples. (B) Photocurrent density and (C) FE_CO_ and FE_H2_ of the Au‐TiO_2_/InP photocathode. Reproduced with permission [94]. Copyright 2021, American Chemical Society. (D) Schematic of the fabrication process, (E) LSV curves, and (F) FE_CO_ and FE_H2_ of the b‐Au_1_/Si photocathode. Reproduced with permission [95]. Copyright 2023, Elsevier. (G) Schematic illustration of the n^+^p^−^Si µW/Cu photocathode. (H) *j*_CH4_ and (I) *j*_C2H4_ of the n^+^p^−^Si µW/Cu photocathode. Reproduced with permission [99]. Copyright 2020, American Chemical Society.

For example, Liu et al. fabricated a photocathode for PEC CO_2_RR to CO by combining InP nanopillar arrays with Au‐TiO_2_ interfaces (Au‐TiO_2_/InP) [94]. In the fabrication process, they used inductively coupled plasma reactive ion etching (ICP‐RIE) on Au/SiO*_x_*‐masked InP wafers to synthesize InP NPs. However, ICP‐RIE led to plasma‐induced surface defects on the InP NPs, resulting in a reduced minority carrier lifetime compared to planar InP. To remedy this, a dilute HCl solution was used to remove the plasma‐damaged layer, leading to fewer surface defects in the InP NPs and improved minority carrier lifetime, as shown in Figure 8A. Thus, these treatments, aimed at eliminating surface defects, contributed to an increased surface area, reduced light reflection, and minimized carrier recombination losses, ultimately enhancing light‐harvesting efficiency. The PEC performance of the nanostructured Au‐TiO_2_/InP photocathodes demonstrated an onset potential of +0.3 V vs. RHE and an FE_CO_ of 84.2% at −0.11 V vs. RHE in a CO_2_‐purged 0.1 m KHCO_3_ solution under simulated 1 sun illumination, as illustrated in Figure 8B,C. Hu et al. developed a photocathode for PEC CO_2_ conversion into CO featuring uniformly dispersed Au NPs with Au (111)/Au (200) boundaries on the p‐Si surface (b‐Au_1_/Si) [95]. Initially, small, consistently sized Au seeds were distributed on the p‐Si surface using a chemical deposition (CD) method. Subsequent electrodeposition ensured continuous growth of the Au above the CD seeds, maintaining a homogeneous distribution, as depicted in Figure 8D. DFT calculations suggested that the presence of Au (111)/Au (200) boundaries substantially decreased the energy barrier for forming the ^*^COOH intermediate during CO_2_RR. As a result, the b‐Au_1_/Si photocathode achieved an impressive photocurrent density of −13.1 mA cm^−2^ at −1.0 V vs. RHE with an FE_CO_ of 82.2% at −0.4 V vs. RHE, and it maintained remarkable operational stability for over a week, as shown in Figure 8E,F. Mubarak et al. prepared a 3D nanoporous structured TiO_2_ NPs on a thin Ti foil photocathode for CO_2_RR to HCOOH; the process involved a chemical treatment with H_2_O_2_, followed by calcination at elevated temperatures ranging from 400 to 800°C [96]. The resulting 300 to 500 nm thick 3D nanoporous layer on the Ti‐foil surface displayed significant porosity. This structure enhanced the photon conversion efficiency of TiO_2_ NPs, increasing photon absorption per unit surface area and strengthening the electrochemical reaction capabilities due to the large specific surface area. The photocathode produced HCOOH as the primary product in PEC CO_2_RR after more than 25 h of chronoamperometric electrolysis and achieved an FE_HCOOH_ of 64% with an HCOOH yield of 165 µmol cm^−2^ h^−1^ at −1.3 V vs. Ag/AgCl. Paul et al. designed a photocathode using a morphology‐controlled synthesis of Ag NPs distributed onto WO_3_ nanorods (Ag/WO_3_‐NR) for PEC CO_2_RR to HCOO^–^ [97]. The one‐pot fabrication process employed cetyltrimethylammonium bromide (CTAB) as a structure‐directing agent for forming WO_3_ nanorods. Critical parameters such as CTAB concentration, reflux time, and temperature were pivotal in determining the nanorod morphology. The Ag/WO_3_‐NR photocathode exhibited a significant current density of 0.4 mA cm^−2^ for HCOO^–^ production and achieved a rate of 31.7 mmol h^−1^.

Gurudayal et al. constructed a photocathode for PEC CO_2_RR to C_2+_ products, utilizing a back‐illuminated n‐type Si absorber covered with an Ag‐supported dendritic Cu catalyst [98]. The synthesis of the Ag‐supported dendritic Cu catalyst involved the evaporation of Ag, followed by high‐rate electrodeposition of Cu, resulting in a highly porous structure with nanocactus‐like morphology and featuring a dendritic Cu structure on the pyramid‐shaped Ag. The Ag‐supported dendritic Cu catalyst exhibited a high electrochemically active surface area, enabling the photocathode to operate at a high current density. Therefore, the photocathode produced C_2+_ products, including C_2_H_4_, C_2_H_5_OH, and C_3_H_7_OH, in CO_2_‐saturated 0.1 m CsHCO_3_ under simulated one sun illumination. Additionally, the photocathode maintained over 60% FE for hydrocarbon and oxygenated products, primarily C_2_H_4_, C_2_H_5_OH, and C_3_H_7_OH, for several days under simulated diurnal illumination. Kempler et al. utilized high loadings of Cu integrated onto Si microwire arrays (n^+^p^−^Si µW/Cu) for PEC CO_2_RR to C_2_H_4_ [99]. The Si microwire array structure could diminish trade‐offs between catalyst loading and light absorption intensity. A Si photocathode with Cu electrodeposited onto the vertical sidewalls of high‐aspect‐ratio microwires was designed to minimize parasitic absorption by the catalyst, as revealed in Figure 8G, demonstrating a |J_ph_| exceeding 25 mA cm^−2^ before and after 48 h of PEC CO_2_RR, resulting in the production of C_2_H_4_ at more positive potentials under 1‐sun illumination. Thus, the n^+^p^−^Si µW/Cu photocathode revealed a maximum |J_C2H4_| of 2.1 ± 0.2 mA cm^−2^ at −0.44 V vs. RHE and a maximum |J_CH4_| of 2.9 ± 0.7 mA cm^−2^ at −0.62 V vs. RHE, as shown in Figure 8H,I.

### 3.4 Defect Engineering

Defect engineering, beyond doping, involves native point defects such as vacancies and interstitials occurring naturally during material synthesis, influencing catalytic, electrical, and optical properties. These defects are now recognized as a strategy to enhance photoactivity in photoelectrode. Classified based on dimensional space, defects include 0D (point defects), 1D (line defects), 2D (interface defects), and 3D (bulk defects) [100, 101, 102, 103, 104]. Their roles encompass both geometric and electronic effects, acting as adsorption sites due to their high energy state and influencing electronic structures. Defects, with their dynamic structures, improve activation and diffusion in stable CO_2_ reactions. Additionally, they alter electronic structures to affect adsorption energy, steering reaction pathways in processes like CO_2_RR. The controlled fabrication of desirable defects is crucial for successful defect engineering, enhancing CO_2_RR kinetics, and minimizing catalysis‐recombination trade‐offs without needing external catalytic entities [105, 106, 107, 108, 109].

**FIGURE 9:**
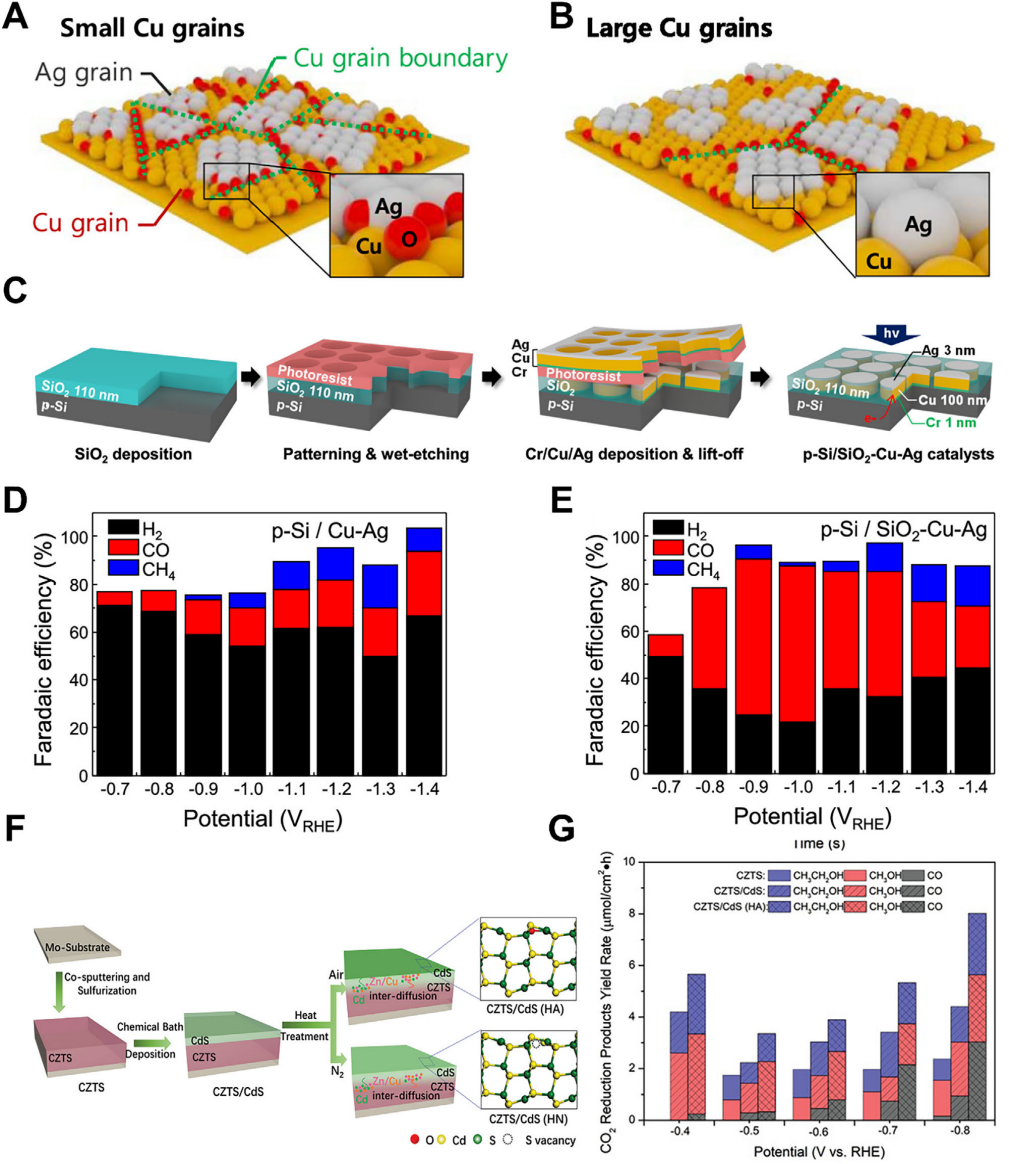
Schematic illustrations of Cu‐Ag thin‐film cocatalyst with (A) small Cu grains and (B) large Cu grains. (C) Schematic depiction of fabrication procedures for the p‐Si / SiO_2_‐Cu‐Ag. FE_total_ from (D) p‐Si/Cu‐Ag and (E) p‐Si / SiO_2_‐Cu‐Ag. Reproduced with permission [110]. Copyright 2021, American Chemical Society. (F) Schematic fabrication processes of CZTS/CdS by different heat treatment conditions (Air or N_2_ atmospheres). (G) CO_2_RR products yield rate of CZTS, CZTS/CdS and CZTS/CdS (HA). Reproduced with permission [113]. Copyright 2021, John Wiley and Sons.

As shown in Figure 9A,B, Dong et al. investigated grain boundary (GB) oxidation in Cu−Ag thin films and its impact on the selectivity of CO and CH_4_ production [110]. They developed a photocathode incorporating a Cu−Ag thin‐film cocatalyst and p‐type Si to enhance the efficiency of PEC CO_2_RR, as revealed in Figure 9C. The electron beam evaporation method facilitated the direct growth of Cu thin films on the substrate, with the grain size easily controlled by adjusting the thickness through nucleation and growth processes. It was observed that oxygen from the surrounding air permeated the Cu thin film through gaps between the Ag islands, leading to the oxidation of the Cu, particularly at the unstable GBs of uncoordinated Cu atoms. Consequently, smaller Cu grains with a higher GB density were prone to oxidation, which compromised the catalytic activity of the Cu−Ag thin‐film catalyst. In contrast, a relatively thick Cu layer (≥80 nm) with a larger grain size effectively prevented oxidation, resulting in catalytic properties comparable to those of bulk Cu−Ag catalysts. Optimizing the Cu (100 nm) − Ag (3 nm) thin‐film catalyst revealed a bifunctional characteristic. This catalyst could selectively produce both CO (FE_CO_ of 79.8%) and CH_4_ (FE_CH4_ of 59.3%) at potentials of −1.0 and −1.4 V vs. RHE, respectively, as shown in Figure 9D,E. Furthermore, introducing a novel PEC architecture comprising the patterned Cu−Ag thin film, a SiO_2_ passivation layer, and a p‐Si photocathode, significantly improved the selectivity of CO and CH_4_ under light illumination (100 mW cm^−2^). Cheng et al. explored the synthesis of CdS NPs featuring controllable S‐vacancies encapsulated within a Zeolitic imidazolate framework‐8 (ZIF‐8) and utilized as a precursor for nitrogen‐doped porous carbon (NCP) through a two‐step process [111]. Initially, CdS NPs were stabilized with polyvinylpyrrolidone (PVP), followed by the deposition of a ZIF‐8 shell onto their surface, resulting in a core‐shell structure with the CdS NPs as the core and ZIF‐8 as the shell. The subsequent control of S vacancies in the CdS and the pyridinic N content in the NCP was achieved through pyrolysis at various temperatures. The CdS/NCP catalysts, serving as cathodes with a TiO_2_ nanotube array photoanode, enhanced the conductivity and stability of TMS‐based catalysts and facilitated CO_2_RR. Based on the pyrolysis temperature, the tunable S vacancies in the CdS/NCP samples led to enhanced selectivity towards CH_3_OH, which is attributed to the synergistic impact of S‐vacancies in CdS and the pyridinic N content in nitrogen‐doped porous carbon. The resulting CdS/NCP material, with its porous structure, abundant S‐vacancies, high pyridinic N content, and enhanced conductivity, proved effective as a cathode catalyst for CO_2_RR. This synergistic effect was precisely controlled by adjusting the temperature (i.e., 300, 500, and 700°C) during the thermal treatment of the hybrid material. Notably, the sample treated at 500°C (CdS/NCP‐500 catalyst) exhibited a high conversion rate (3052 nmol·h^−1^·cm^−2^) with a selectivity of 77.3% towards CH_3_OH.

Kan et al. ingeniously devised and crafted a p‐Si/n‐ZnO_v_/p‐Cu*_x_* O heterostructure, incorporating a ZnO*_v_*‐derived Cu*_x_* O defect level that exhibits a remarkable capability for selective PEC CO_2_RR to C_2_H_5_OH at low biases [112]. The p‐n‐p band alignment was pivotal in confining and accumulating multiple electrons within the conduction band of n‐type ZnO_v_, facilitated by a built‐in electric field. Simultaneously, the shallow ZnO_v_ defect level (vE_ZnOv_) allowed electrons to escape from the confined well and reach the Cu*_x_* O (E_Cu_ *_x_*_O_, 0.05 V vs. RHE). These tunneling defect energy levels on Cu*_x_* O closely aligned with those required for the CO_2_ to C_2_H_5_OH reduction, contributing to the heterostructure's exceptional selectivity for PEC CO_2_RR towards C_2_H_5_OH at low biases, accompanied by an outstanding FE. In contrast, control samples, including p‐Si/p‐Cu*_x_* O and p‐Si/n‐ZnO_v_, necessitated higher overpotentials to overcome larger energy barriers, resulting in distinct CO_2_RR selectivity towards CH_4_ and HCOO^–^, respectively. The transfer of photoelectrons in the Si/ZnO_v_/Cu*_x_* O system was facilitated by a built‐in electric field of approximately 0.6 V through a leaky tunnel formed in the defect levels of ZnO_v_ and Cu*_x_* O, allowing for a close matching of energy levels and enabling the selective conversion of CO_2_ into C_2_H_5_OH. The optimized potential and functional interface contributed to achieving an impressive FE exceeding 60% for PEC reduction of CO_2_ to C_2_H_5_OH under 0 V vs. RHE. In addition to the prerequisites for efficient charge carrier transfer, studies have indicated that defect engineering plays a crucial role in regulating catalytic activity and CO_2_RR selectivity based on the surface state of the photocatalyst. Nevertheless, the specific impact of sulfur vacancies on the transfer of photogenerated e^–^/h^+^ pairs and the reaction mechanism during CO_2_RR remains unclear. Zhou et al. implemented a heat treatment strategy on Cu_2_ZnSnS_4_/CdS (CZTS/CdS) photocathodes, achieving simultaneous optimization of interface charge transfer and surface S vacancy engineering [113]. These advancements significantly contributed to the enhanced overall PEC CO_2_RR performance and manageable selectivity. Heat treatment improved the CZTS/CdS heterojunction interface by promoting elemental inter‐diffusion between Cd in CdS and Cu/Zn in CZTS, as shown in Figure 9F. This resulted in a more favorable p‐n junction with an enlarged built‐in potential, prolonged carrier lifetime, and suppressed charge recombination. Additionally, defects on the surface of CdS could be modulated through heat treatment in different atmospheres. Heat treatment in air replenished intrinsic S vacancies on the CZTS/CdS surface with oxygen, enhancing CO_2_ and CO adsorption capability, as observed in Figure 9G, which leads to improved CO_2_RR activity and higher selectivity toward CH_3_OH/C_2_H_5_OH. Conversely, heat treatment in N_2_ generated more S vacancies on the surface, facilitating surficial CO desorption and higher CO selectivity. By combining heterojunction design and modification of catalyst surface properties through a simple heat treatment strategy, this work established a new approach to designing photocathodes for high‐performance PEC CO_2_RR activity with controlled selectivity. Table 2 lists the performance of interface‐engineered photocathodes for PEC CO_2_RR.

**TABLE 2:** Summary of the strategy for interface engineering of a photocathode for PEC CO_2_RR.

Strategy	Photocathode materials	Electrolyte	Performance about activity and selectivity	Ref.
Plasmonic noble metal cocatalysts	CuBi_2_O_4_ IOs‐Ag	0.1 m KHCO_3_	FE_CO_ of 92%	[48]
	Au/TiO_2_/n^+^p^–^ Si	0.1 m KHCO_3_	*j*_CO_ of 5.52 mA cm^−2^, FE_CO_ of 86%	[49]
	Ag‐TiO_2_/RGO	1.0 m KOH	*j*_total_ of 23.5 mA cm^−2^, FE_CH3OH_ of 60.5%	[50]
	Au/α‐Fe_2_O_3_/RGO	0.1 m KOH	*j_t_*_otal_ of −31.5 mA cm^−2^, FE_CH3OH_ of 21.5%	[51]
	Ag/Cu‐TS‐1	0.1 m KHCO_3_	C_2_H_5_OH yield rate of 2.62 µmol cm^−2^ h^−1^	[52]
	Cu_2_O/Ag	0.1 m KHCO_3_	Yield rate of 212.7 µmol cm^−2^ h^−1^, FE_CH3COOH_ of 47.7%	[53]
Non‐noble metal cocatalysts	STA‐GO/CoPc	0.1 m KHCO_3_	*j*_total_ of 0.7 mA cm^−2^, FE_CO_ of 86%, FE_CH3OH_ of 8%	[62]
	Si|mesoTiO_2_|CoPcP	0.5 m KHCO_3_	TONco of 939 ± 132, FE_CO_ of 66%	[63]
	Si|TiO_2_|CNT|Co^II^(BrqPy)	0.1 m KHCO_3_	*j*_total_ of −1.4 mA cm^−2^, FE_CO_ of 97%	[64]
	Si|mesoTiO_2_|CotpyP	MeCN with 0.1 m TBABF_4_	TON of 381	[65]
	CuS/GaN/Si	0.1 m KHCO_3_	*j*_HCOOH_ of 7.07 mA cm^−2^, FE_HCOOH_ of 70.2%	[66]
	Bi/GaN/Si	0.1 m KHCO_3_	*j*_HCOOH_ of 10.3 mA cm^−2^, FE_HCOOH_ of 98%	[67]
	Sn NP/GaN NW/Si	0.1 m KHCO_3_	*j*_total_ of 17.5 mA cm^−2^, FE_HCOOH_ of 76.9%	[68]
	Cu_3_(BTC)_2_/Cu_2_O	MeCN with 0.1 m TBAPF_6_	FE_CO_ of about 95%	[69]
	CuN*_x_*/CuO	0.1 m KHCO_3_	*j*_total_ of −1.0 mA cm^−2^, FE_C2_ of 15.2%	[70]
	Cu NPs/Si NWs	0.1 m KHCO_3_	*j*_C2H4_ exceeding 2.5 mA cm^−2^, FE_C2H4_ of about 25%	[71]
	Cu/TiO_2_/p‐Si	0.1 m CsHCO_3_	*j*_C2H4_ of −2.3 mA cm^−2^	[72]
Junction engineering	Bi@ZFO NTs	0.1 m KHCO_3_	FE_HCOOH_ of 61.2%	[74]
	CuO/g‐C_3_N_4_/carbon paper	0.1 m NaHCO_3_	FE_CH3OH_ of 75%	[75]
	CeO_2_ NPs/CuO NPs/Cu	0.1 m KHCO_3_	CH_3_OH yield rate of 3.44 µmol cm^−2^ h^−1^, FE_CH3OH_ of 60%	[76]
	ZnPc/carbon nitride	0.1 m KHCO_3_	CH_3_OH yield of 13 µmol L^−1^	[77]
	CdS‐CuFe_2_O_4_	0.1 m NaHCO_3_	CH_3_OH yield of 23.8 µmol L^−1^ cm^−2^, FE_CH3OH_ of 72%	[78]
	Pd@TiO_2_/Ti_3_CN	0.1 m KHCO_3_	yield rate of 45.6 µM cm^−2^ h^−1^	[79]
	CuFeO_2_/CuO nanowire	Triethanolamine	FE_C2H5OH_ of 66.73%	[80]
	CuFeO_2_/TNNTs	0.1 m NaHCO_3_	*j*_total_ of 80 µA cm^−2^, C_2_H_5_OH yield rate of 3.3 µmol/5h·cm^−2^	[81]
	BCW‐6	0.1 m KHCO_3_	C_2_H_5_OH yield rate of 600 µmol h^−1^ g^−1^, FE_C2H5OH_ of 80%	[82]
	g‐C_3_N_4_/ZnTe	0.1 m KHCO_3_	C_2_H_5_OH yield rate of 17.1 µmol cm^−2^ h^−1^	[83]
	Zn*_x_*:Co*_y_* @Cu	0.1 m KHCO_3_	paraffin yield rate of 325 µg h^−1^	[84]
Nanosturcture engineering	Au‐TiO_2_/InP	0.1 m KHCO_3_	FE_CO_ of 84.2%	[94]
	b‐Au_1_/Si	0.1 m KHCO_3_	*j*_total_ of −13.1 mA cm^−2^, FE_CO_ of 82.2%	[95]
	TO600	1 m KOH	HCOOH yield rate of 165 µmol cm^−2^ h^−1^, FE_HCOOH_ of 64%	[96]
	Ag/WO_3_‐NR	0.1 m KHCO_3_	*j*_HCOO–_ of 0.4 mA cm^−2^, HCOO^–^ yield rate of 31.7 mmol h^−1^	[97]
	Ag‐supported dendritic Cu	0.1 m CsHCO_3_	FE_C2+_ of 60%	[98]
	n^+^p^−^Si µW/Cu	0.1 m KHCO_3_	*j*_CH4_ of 2.9 ± 0.7 mA cm^−2^, *j*_C2H4_ of 2.1 ± 0.2 mA cm^−2^	[99]
Defect engineering	Cu−Ag thin film	0.1 m KHCO_3_	FE_CO_ of 79.8%, FE_CH4_ of 59.3%	[110]
	CdS/NCP	0.5 m KHCO_3_	FE_CH3OH_ of 77.3%	[111]
	p‐Si/n‐ZnO_v_/p‐Cu*_x_* O	0.1 m KHCO_3_	FE_C2H5OH_ of exceeding 60%	[112]
	CZTS/CdS	0.1 m KHCO_3_	CO yield rate of 2.31 µmol cm^−2^ h^−1^	[113]

## 4 Conclusions and Perspectives

This review underscores the critical role of interface engineering in optimizing the efficiency of PEC photocathode, providing valuable insights into recent research trends, and contributing to the global pursuit of carbon neutrality. Interface engineering emerges as a pivotal strategy, optimizing cocatalysts, junction engineering, nanostructure engineering, and defect engineering to overcome existing limitations. Noble metal cocatalysts, such as Ag and Au, are seamlessly integrated with plasmonic materials to induce surface plasmon resonance, thereby enhancing light absorption and catalytic efficiency for PEC CO_2_RR. This enhancement is evident in various studies utilizing diverse nanostructure and synthesis methods of plasmonic noble metal cocatalysts. Moreover, when semiconductor materials are integrated with various non‐noble metal cocatalysts, they demonstrate promising advancements in enhancing electrocatalytic activity and selectivity for PEC CO_2_RR. Semiconductor heterojunctions, encompassing type‐I, type‐II, and Schottky barrier junctions, play a pivotal role in enhancing PEC CO_2_RR by facilitating the separation of photogenerated electrons and holes through distinct band structures. Nanostructure engineering further augments photocathode performance by manipulating materials on the nanometer scale, employing structures such as nanorods, nanowires, and core/shell configurations. Defect engineering, involving native point defects like vacancies and interstitials, influences catalytic, electrical, and optical properties. These defects, classified as 0D (point defects), 1D (line defects), 2D (interface defects), and 3D (bulk defects), enhance photoactivity in photocathode for PEC CO_2_RR. Despite the numerous research outcomes, interface engineering still requires further studies. These studies should address the following issues:

- The PEC process based on interface engineering often involves complex procedures and expensive equipment, necessitating the development of more streamlined and cost‐effective approaches.
- Extensive research is required to assess the long‐term performance, stability, and durability of photocathode used in PEC CO_2_RR experiments, particularly in challenging environmental conditions.
- Further research is required at the single photoelectrode level and beyond to achieve the ultimate goal of harnessing sunlight directly, without the need for additional external bias.
- Current investigations into PEC CO_2_RR predominantly yield C_1_ products; therefore, a critical need exists for comprehensive studies focusing on C_2+_ products, which offer higher value‐added potential.

Researchers have made significant strides in addressing these challenges. Notably, the interface engineering of photocathode has demonstrated potential in improving PEC CO_2_RR system performance by effectively capturing and segregating photogenerated charge carriers while minimizing recombination. Nevertheless, the implementation of interface engineering continues to present diverse challenges, including the complexity of the process, the necessity for stability, the requirement to advance to the device level, and the limited selectivity of the products. To achieve successful utilization of the PEC CO_2_RR system, it is essential to develop a more sophisticated and facile interface engineering technique that can effectively address the aforementioned challenges. The incorporation of suitable interlayers, transport layers, and electron‐blocking layers is essential for enhancing stability of photocathode. The design of efficient configurations with multijunction photocathodes represents an effective means of enhancing photocathode utilization and overall efficiency. The cocatalysts play an electrochemically pivotal role in promoting desired PEC CO_2_RR, with Cu cocatalysts being particularly effective in producing C_2+_ products. The integration of these cocatalysts into composite materials and the modification of their structure can facilitate the production of various C_2+_ products.

In conclusion, this review paper examines the current trends of research on photocathode for PEC CO_2_RR from the perspective of interface engineering. By identifying these trends, the paper aims to make a significant contribution to the broader field of CO_2_RR research.

## Conflicts of Interest Statement

The authors declare no conflicts of interest.

## References

[1] T. R. Anderson , E. Hawkins , and P. D. Jones , “CO_2_, the Greenhouse Effect and Global Warming: From the Pioneering Work of Arrhenius and Callendar to Today's Earth System Models,” Endeavour 40 (2016): 178. 27469427 10.1016/j.endeavour.2016.07.002

[2] Y. Xie , T. T. Wang , X. H. Liu , K. Zou , and W. Q. Deng , “Capture and Conversion of CO_2_ at Ambient Conditions by a Conjugated Microporous Polymer,” Nature Communications 4 (2013): 1960. 10.1038/ncomms2960 PMC3709476 23727768

[3] S. Xiang , Y. He , Z. Zhang , et al., “Microporous Metal‐organic Framework With Potential for Carbon Dioxide Capture at Ambient Conditions,” Nature Communications 3 (2012): 954. 10.1038/ncomms1956 22805561

[4] Z. Liu , Z. Deng , B. Zhu , et al., “Global Patterns of Daily CO_2_ Emissions Reductions in the First Year of COVID‐19,” Nature Geoscience 15 (2022): 615.

[5] V. Masson‐Delmotte , P. Zhai , A. Pirani , et al., IPCC, 2021: Climate Change 2021: The Physical Science Basis. Contribution of Working Group I to the Sixth Assessment Report of the Intergovernmental Panel on Climate Change (Cambridge University Press, 2021), 2391.

[6] Z. Liu , Z. Deng , S. Davis , and P. Ciais , “Monitoring Global Carbon Emissions in 2022,” Nature Reviews Earth & Environment 4 (2023): 205. 10.1038/s43017-023-00406-z PMC10010646 37065615

[7] Y. R. Wang , Q. Huang , C. T. He , et al., “Oriented Electron Transmission in Polyoxometalate‐Metalloporphyrin Organic Framework for Highly Selective Electroreduction of CO_2_ ,” Nature Communications 9 (2018): 4466. 10.1038/s41467-018-06938-z PMC6203756 30367039

[8] N. T. Nesbitt , M. Ma , B. J. Trześniewski , et al., “Au Dendrite Electrocatalysts for CO_2_ Electrolysis,” Journal of Physical Chemistry C 122 (2018): 10006.

[9] Y. T. Guntern , J. R. Pankhurst , J. Vavra , et al., “Nanocrystal/Metal–Organic Framework Hybrids as Electrocatalytic Platforms for CO_2_ Conversion,” Angewandte Chemie 131 (2019): 12762. 10.1002/anie.201905172 31287203

[10] L. K. Putri , W. J. Ong , W. S. Chang , and S. P. Chai , “Enhancement in the Photocatalytic Activity of Carbon Nitride Through Hybridization With Light‐Sensitive AgCl for Carbon Dioxide Reduction to Methane,” Catalysis Science & Technology 6 (2016): 744.

[11] X. Y. Kong , T. Tong , B. J. Ng , et al., “Topotactic Transformation of Bismuth Oxybromide Into Bismuth Tungstate: Bandgap Modulation of Single‐Crystalline {001}‐Faceted Nanosheets for Enhanced Photocatalytic CO_2_ Reduction,” ACS Appl Mater Interfaces 12 (2020): 26991. 32433865 10.1021/acsami.9b15950

[12] X. Y. Kong , Y. Y. Choo , S. P. Chai , A. K. Soh , and A. R. Mohamed , “Oxygen Vacancy Induced Bi 2 WO 6 for the Realization of Photocatalytic CO_2_ Reduction Over the Full Solar Spectrum: From the UV to the NIR Region,” Chemical Communications 52 (2016): 14242. 27872917 10.1039/c6cc07750a

[13] X. Y. Kong , W. L. Tan , B. J. Ng , S. P. Chai , and A. R. Mohamed , “Harnessing Vis–NIR Broad Spectrum for Photocatalytic CO_2_ Reduction Over Carbon Quantum Dots‐decorated Ultrathin Bi2WO6 Nanosheets,” Nano Research 10 (2017): 1720.

[14] Z. Yang , Y. Qi , F. Wang , et al., “State‐of‐the‐art Advancements in Photo‐assisted CO_2_ Hydrogenation: Recent Progress in Catalyst Development and Reaction Mechanisms,” Journal of Materials Chemistry A 8 (2020): 24868.

[15] T. Kulandaivalu , A. R. Mohamed , K. A. Ali , and M. Mohammadi , “Photocatalytic Carbon Dioxide Reforming of Methane as an Alternative Approach for Solar Fuel Production‐a Review,” Renewable & Sustainable Energy Reviews 134 (2020): 110363.

[16] T. Amrillah , A. R. Supandi , V. Puspasari , A. Hermawan , and Z. W. Seh , “MXene‐Based Photocatalysts and Electrocatalysts for CO_2_ Conversion to Chemicals,” Transactions of Tianjin University 28 (2022): 307.

[17] J. H. Cho , J. Ma , and S. Y. Kim , “Toward High‐Efficiency Photovoltaics‐Assisted Electrochemical and Photoelectrochemical CO_2_ Reduction: Strategy and Challenge,” Exploration 3 (2023): 20230001. 37933280 10.1002/EXP.20230001 PMC10582615

[18] D. Yang , H. Yu , T. He , et al., “Visible‐light‐switched Electron Transfer Over Single Porphyrin‐metal Atom Center for Highly Selective Electroreduction of Carbon Dioxide,” Nature Communications 10 (2019): 3844. 10.1038/s41467-019-11817-2 PMC6710284 31451689

[19] G. H. Han , J. Bang , G. Park , et al., “Recent Advances in Electrochemical, Photochemical, and Photoelectrochemical Reduction of CO_2_ to C2+ Products,” Small 19 (2023): 2205765. 10.1002/smll.202205765 36592422

[20] R. Kortlever , J. Shen , K. J. P. Schouten , F. Calle‐Vallejo , and M. T. Koper , “Catalysts and Reaction Pathways for the Electrochemical Reduction of Carbon Dioxide,” Journal of Physical Chemistry Letters 6 (2015): 4073. 26722779 10.1021/acs.jpclett.5b01559

[21] J. T. Feaster , C. Shi , E. R. Cave , et al., “Understanding Selectivity for the Electrochemical Reduction of Carbon Dioxide to Formic Acid and Carbon Monoxide on Metal Electrodes,” ACS Catalysis 7 (2017): 4822.

[22] A. Bagger , W. Ju , A. S. Varela , P. Strasser , and J. Rossmeisl , “Electrochemical CO_2_ Reduction: A Classification Problem,” Chemphyschem 18 (2017): 3266. 28872756 10.1002/cphc.201700736

[23] J. Santatiwongchai , K. Faungnawakij , and P. Hirunsit , “Comprehensive Mechanism of CO_2_ Electroreduction Toward Ethylene and Ethanol: The Solvent Effect From Explicit Water–Cu(100) Interface Models,” ACS Catalysis 11 (2021): 9688.

[24] M. Halmann , “Photoelectrochemical Reduction of Aqueous Carbon Dioxide on p‐Type Gallium Phosphide in Liquid Junction Solar Cells,” Nature 275 (1978): 115.

[25] V. H. Nguyen , B. S. Nguyen , Z. Jin , et al., “Towards Artificial Photosynthesis: Sustainable Hydrogen Utilization for Photocatalytic Reduction of CO_2_ to High‐Value Renewable Fuels,” Journal of Chemical Engineering 402 (2020): 126184.

[26] T. P. Nguyen , D. L. T. Nguyen , V. H. Nguyen , et al., “Recent Advances in TiO_2_‐Based Photocatalysts for Reduction of CO_2_ to Fuels,” Nanomaterials 10 (2020): 337. 32079215 10.3390/nano10020337 PMC7075154

[27] H. H. Do , D. L. T. Nguyen , X. C. Nguyen , et al., “Recent Progress in TiO2‐based Photocatalysts for Hydrogen Evolution Reaction: A Review,” Arabian Journal of Chemistry 13 (2020): 3653.

[28] S. Thiele , J. Bachmann , and S. Cherevko , “Dissolution of WO_3_ Modified With IrOx Overlayers During Photoelectrochemical Water Splitting,” SusMat 3 (2023): 128.

[29] P. Ding , T. Jiang , N. Han , and Y. Li , “Photocathode Engineering for Efficient Photoelectrochemical CO_2_ Reduction,” Materials Today Nano 10 (2020): 100077.

[30] W. Zhang , Z. Jin , and Z. Chen , “Rational‐Designed Principles for Electrochemical and Photoelectrochemical Upgrading of CO_2_ to Value‐Added Chemicals,” Advancement of Science 9 (2022): 2105204. 10.1002/advs.202105204 PMC8948570 35072349

[31] A. U. Pawar , C. W. Kim , M.‐T. Nguyen‐Le , and Y. S. Kang , “General Review on the Components and Parameters of Photoelectrochemical System for CO_2_ Reduction With In Situ Analysis,” ACS Sustainable Chemistry & Engineering 7 (2019): 7431.

[32] Y. Deng and B. S. Yeo , “Characterization of Electrocatalytic Water Splitting and CO_2_ Reduction Reactions Using In Situ/Operando Raman Spectroscopy,” ACS Catalysis 7 (2017): 7873.

[33] M. J. Kang , C. W. Kim , A. U. Pawar , et al., “Selective Alcohol on Dark Cathodes by Photoelectrochemical CO_2_ Valorization and Their In Situ Characterization,” ACS Energy Letters 4 (2019): 1549.

[34] H. Gong , Z. Wei , Z. Gong , et al., “Low‐Coordinated Co‐N‐C on Oxygenated Graphene for Efficient Electrocatalytic H_2_O_2_ Production,” Advanced Functional Materials 32 (2022): 2106886.

[35] J. Ma , J. H. Cho , C. Lee , et al., “Unraveling the Harmonious Coexistence of Ruthenium States on a Self‐Standing Electrode for Enhanced Hydrogen Evolution Reaction,” Energy & Environmental Materials 7 (2024): e12766.

[36] Y. Sun , S. Ding , B. Xia , J. Duan , M. Antonietti , and S. Chen , “Biomimetic FeMo(Se, Te) as Joint Electron Pool Promoting Nitrogen Electrofixation,” Angewandte Chemie 134 (2022): e202115198. 10.1002/anie.202115198 35076985

[37] A. Molinari , L. Samiolo , and R. Amadelli , “EPR Spin Trapping Evidence of Radical Intermediates in the Photo‐reduction of Bicarbonate/CO_2_ in TiO_2_ Aqueous Suspensions,” Photochemical & Photobiological Sciences 14 (2015): 1039. 25849227 10.1039/c4pp00467a

[38] J. Ma , S. H. Ahn , and S. Y. Kim , “Integration of Earth‐abundant Cocatalysts for High‐performance Photoelectrochemical Energy Conversion,” Journal of Energy Chemistry 88 (2024): 336.

[39] J. Liu , C. Xia , S. Zaman , Y. Su , L. Tan , and S. Chen , “Surface Plasmon Assisted Photoelectrochemical Carbon Dioxide Reduction: Progress and Perspectives,” Journal of Materials Chemistry A 11 (2023): 16918.

[40] K. Watanabe , D. Menzel , N. Nilius , and H. J. Freund , “Photochemistry on Metal Nanoparticles,” Chemical Reviews 106 (2006): 4301. 17031988 10.1021/cr050167g

[41] S. Linic , P. Christopher , and D. B. Ingram , “Plasmonic‐metal Nanostructures for Efficient Conversion of Solar to Chemical Energy,” Nature Materials 10 (2011): 911. 22109608 10.1038/nmat3151

[42] G. Baffou and R. Quidant , “Nanoplasmonics for Chemistry,” Chemical Society Reviews 43 (2014): 3898. 24549257 10.1039/c3cs60364d

[43] U. Aslam , V. G. Rao , S. Chavez , and S. Linic , “Catalytic Conversion of Solar to Chemical Energy on Plasmonic Metal Nanostructures,” Nature Catalysis 1 (2018): 656.

[44] S. Chen , W. H. Li , W. Jiang , et al., “MOF Encapsulating N‐Heterocyclic Carbene‐Ligated Copper Single‐Atom Site Catalyst towards Efficient Methane Electrosynthesis,” Angewandte Chemie International Edition 134 (2022): e202114450. 10.1002/anie.202114450 34767294

[45] V. H. Nguyen , B. S. Nguyen , and C. C. Hu , “Novel Architecture Titanium Carbide (Ti3C2Tx) MXene Cocatalysts Toward Photocatalytic Hydrogen Production: A Mini‐Review,” Nanomaterials 10 (2020): 602. 32218204 10.3390/nano10040602 PMC7221605

[46] S. Bae , S. Lee , H. Ryu , and W. J. Lee , “Improvement of Photoelectrochemical Properties of CuO Photoelectrode by Li Doping,” Korean Journal of Metals and Materials 60 (2022): 577.

[47] V. Kumar , R. K. Mishra , L. G. Trung , et al., “Copper, Palladium, and Reduced Graphene Oxide co‐doped Layered WS2/WO3 Nanostructures for Electrocatalytic Hydrogen Generation,” Electronic Materials Letters 20 (2024): 414.

[48] G. Liu , R. Cai , Z. Lv , et al., “Ameliorating the Carrier Dynamics Behavior via Plasmonic Ag‐modified CuBi_2_O_4_ Inverse Opal for the Efficient Photoelectrocatalytic Reduction of CO_2_ to CO,” Journal of Catalysis 424 (2023): 130.

[49] K. Wang , N. Fan , B. Xu , et al., “Steering the Pathway of Plasmon‐Enhanced Photoelectrochemical CO_2_ Reduction by Bridging Si and Au Nanoparticles Through a TiO_2_ Interlayer,” Small 18 (2022): 2201882. 10.1002/smll.202201882 35435325

[50] G. Bharath , J. Prakash , K. Rambabu , et al., “Synthesis of TiO_2_/RGO With Plasmonic Ag Nanoparticles for Highly Efficient Photoelectrocatalytic Reduction of CO_2_ to Methanol Toward the Removal of an Organic Pollutant From the Atmosphere,” Environmental Pollution 281 (2021): 116990. 33812129 10.1016/j.envpol.2021.116990

[51] G. Bharath , K. Rambabu , A. Hai , et al., “Dual‐functional Paired Photoelectrocatalytic System for the Photocathodic Reduction of CO_2_ to Fuels and the Anodic Oxidation of Furfural to Value‐Added Chemicals,” Applied Catalysis B 298 (2021): 120520.

[52] G. Li , M. Wang , H. Shao , et al., “Light‐Driven Carbon Dioxide Reduction Over the Ag‐Decorated Modified TS‐1 Zeolite,” Catalysis Science & Technology 12 (2022): 2490.

[53] Y. Zhang , Q. Wang , K. Wang , et al., “Plasmonic Ag‐decorated Cu_2_O Nanowires for Boosting Photoelectrochemical CO_2_ Reduction to Multi‐Carbon Products,” Chemical Communications 58 (2022): 9421. 35916216 10.1039/d2cc03167a

[54] L. K. Putri , B. J. Ng , W. J. Ong , S. P. Chai , and A. R. Mohamed , “Toward Excellence in Photocathode Engineering for Photoelectrochemical CO_2_ Reduction: Design Rationales and Current Progress,” Advanced Energy Materials 12 (2022): 2201093.

[55] J. C. Matsubu , E. T. Lin , K. L. Gunther , K. N. Bozhilov , Y. Jiang , and P. Christopher , “Critical Role of Interfacial Effects on the Reactivity of Semiconductor‐cocatalyst Junctions for Photocatalytic Oxygen Evolution From Water,” Catalysis Science & Technology 6 (2016): 6836.

[56] C. Shi , H. A. Hansen , A. C. Lausche , and J. K. Nørskov , “Trends in Electrochemical CO_2_ Reduction Activity for Open and Close‐packed Metal Surfaces,” Physical Chemistry Chemical Physics 16 (2014): 4720. 24468980 10.1039/c3cp54822h

[57] Y. Sun , X. Liu , M. Zhu , et al., “Non‐Noble Metal Single Atom‐Based Catalysts for Electrochemical Reduction of CO_2_: Synthesis Approaches and Performance Evaluation,” DeCarbon 2 (2023): 100018.

[58] P. Rao , Y. Yu , S. Wang , et al., “Understanding the Improvement Mechanism of Plasma Etching Treatment on Oxygen Reduction Reaction Catalysts,” Exploration 4 (2024): 20230034. 38854495 10.1002/EXP.20230034 PMC10867369

[59] S. K. Choi , U. Kang , S. Lee , D. J. Ham , S. M. Ji , and H. Park , “Sn‐Coupled p‐Si Nanowire Arrays for Solar Formate Production From CO_2_ ,” Advanced Energy Materials 4 (2014): 1301614.

[60] Q. Shen , Z. Chen , X. Huang , M. Liu , and G. Zhao , “High‐Yield and Selective Photoelectrocatalytic Reduction of CO_2_ to Formate by Metallic Copper Decorated Co_3_O_4_ Nanotube Arrays,” Environmental Science & Technology 49 (2015): 5828. 25844931 10.1021/acs.est.5b00066

[61] K. Alenezi , S. K. Ibrahim , P. Li , and C. J. Pickett , “Solar Fuels: Photoelectrosynthesis of CO From CO_2_ at p‐Type Si Using Fe Porphyrin Electrocatalysts,” Chemistry – A European Journal 19 (2013): 13522. 23946131 10.1002/chem.201300764

[62] B. Shang , C. L. Rooney , D. J. Gallagher , et al., “Aqueous Photoelectrochemical CO_2_ Reduction to CO and Methanol Over a Silicon Photocathode Functionalized With a Cobalt Phthalocyanine Molecular Catalyst,” Angewandte Chemie 135 (2023): e202215213. 10.1002/anie.202215213 36445830

[63] S. Roy , M. Miller , J. Warnan , J. J. Leung , C. D. Sahm , and E. Reisner , “Electrocatalytic and Solar‐Driven Reduction of Aqueous CO_2_ With Molecular Cobalt Phthalocyanine–Metal Oxide Hybrid Materials,” ACS Catalysis 11 (2021): 1868.

[64] Z. Wen , S. Xu , Y. Zhu , et al., “Aqueous CO_2_ Reduction on Si Photocathodes Functionalized by Cobalt Molecular Catalysts/Carbon Nanotubes,” Angewandte Chemie 134 (2022): e202201086. 10.1002/anie.202201086 35225405

[65] J. J. Leung , J. Warnan , K. H. Ly , et al., “Solar‐Driven Reduction of Aqueous CO_2_ With a Cobalt Bis(Terpyridine)‐Based Photocathode,” Nat Catalysis 2 (2019): 354.

[66] W. J. Dong , I. A. Navid , Y. Xiao , J. W. Lim , J. L. Lee , and Z. Mi , “CuS‐Decorated GaN Nanowires on Silicon Photocathodes for Converting CO_2_ Mixture Gas to HCOOH,” Journal of the American Chemical Society 143 (2021): 10099. 34210119 10.1021/jacs.1c02139

[67] W. J. Dong , I. A. Navid , Y. Xiao , et al., “Bi Catalysts Supported on GaN Nanowires Toward Efficient Photoelectrochemical CO_2_ Reduction,” Journal of Materials Chemistry A 10 (2022): 7869.

[68] B. Zhou , X. Kong , S. Vanka , et al., “A GaN:Sn Nanoarchitecture Integrated on a Silicon Platform for Converting CO_2_ to HCOOH by Photoelectrocatalysis,” Energy & Environmental Science 12 (2019): 2842.

[69] X. Deng , R. Li , S. Wu , et al., “Metal–Organic Framework Coating Enhances the Performance of Cu_2_O in Photoelectrochemical CO_2_ Reduction,” Journal of the American Chemical Society 141 (2019): 10924. 31200598 10.1021/jacs.9b06239

[70] K. Wang , Y. Liu , Q. Wang , et al., “Asymmetric Cu‐N Sites on Copper Oxide Photocathode for Photoelectrochemical CO_2_ Reduction towards C_2_ Products,” Applied Catalysis B 316 (2022): 121616.

[71] I. Roh , S. Yu , C. K. Lin , S. Louisia , S. Cestellos‐Blanco , and P. Yang , “Photoelectrochemical CO_2_ Reduction Toward Multicarbon Products With Silicon Nanowire Photocathodes Interfaced With Copper Nanoparticles,” Journal of the American Chemical Society 144 (2022): 8002. 35476928 10.1021/jacs.2c03702

[72] C. Kim , A. J. King , S. Aloni , F. M. Toma , A. Z. Weber , and A. T. Bell , “Codesign of an Integrated Metal–Insulator–Semiconductor Photocathode for Photoelectrochemical Reduction of CO_2_ to Ethylene,” Energy & Environmental Science 16 (2023): 2968.

[73] L. Liu , Y. Zhang , and H. Huang , “Junction Engineering for Photocatalytic and Photoelectrocatalytic CO_2_ Reduction,” Solar RRL 5 (2021): 2000430.

[74] T. Ouyang , Y. Q. Ye , C. Tan , et al., “1D α‐Fe_2_O_3_ /ZnO Junction Arrays Modified by Bi as Photocathode: High Efficiency in Photoelectrochemical Reduction of CO_2_ to HCOOH,” Journal of Physical Chemistry Letters 13 (2022): 6867. 35861318 10.1021/acs.jpclett.2c01509

[75] X. X. Jiang , X. De Hu , M. Tarek , et al., “Tailoring the Properties of G‐C3N4 With CuO for Enhanced Photoelectrocatalytic CO_2_ Reduction to Methanol,” Journal of CO2 Utilization 40 (2020): 101222.

[76] Z. Pan , E. Han , J. Zheng , et al., “Highly Efficient Photoelectrocatalytic Reduction of CO_2_ to Methanol by a p‐n Heterojunction CeO_2_/CuO/Cu Catalyst,” Nano‐Micro Letters 12 (2020): 18. 34138070 10.1007/s40820-019-0354-1 PMC7770658

[77] J. Zheng , X. Li , Y. Qin , et al., “Zn Phthalocyanine/Carbon Nitride Heterojunction for Visible Light Photoelectrocatalytic Conversion of CO_2_ to Methanol,” Journal of Catalysis 371 (2019): 214.

[78] M. Tarek , K. M. R. Karim , S. M. Sarkar , et al., “Hetero‐structure CdS–CuFe_2_O_4_ as an Efficient Visible Light Active Photocatalyst for Photoelectrochemical Reduction of CO_2_ to Methanol,” International Journal of Hydrogen Energy 44 (2019): 26271.

[79] Y. Xu , F. Wang , S. Lei , et al., “In Situ Grown Two‐dimensional TiO_2_/Ti_3_CN MXene Heterojunction Rich in Ti^3+^ Species for Highly Efficient Photoelectrocatalytic CO_2_ Reduction,” Journal of Chemical Engineering 452 (2023): 139392.

[80] M. Lu , D. Jia , H. Xue , J. Tian , and T. Jiang , “0D/1D CuFeO_2_/CuO Nanowire Heterojunction Arrays for Improved Photoelectrocatalytic Reduction of CO_2_ to Ethanol,” Journal of Alloys and Compounds 960 (2023): 170626.

[81] L. Zhang , H. Cao , Y. Lu , et al., “Effective Combination of CuFeO_2_ With High Temperature Resistant Nb‐doped TiO_2_ Nanotube Arrays for CO_2_ Photoelectric Reduction,” Journal of Colloid & Interface Science 568 (2020): 198. 32088450 10.1016/j.jcis.2020.01.082

[82] J. Wang , Y. Wei , B. Yang , B. Wang , J. Chen , and H. Jing , “In Situ Grown Heterojunction of Bi_2_WO_6_/BiOCl for Efficient Photoelectrocatalytic CO_2_ Reduction,” Journal of Catalysis 377 (2019): 209.

[83] Q. Wang , X. Wang , Z. Yu , et al., “Artificial Photosynthesis of Ethanol Using Type‐II G‐C3N_4_/ZnTe Heterojunction in Photoelectrochemical CO_2_ Reduction System,” Nano Energy 60 (2019): 827.

[84] J. Wang , Y. Guan , X. Yu , et al., “Photoelectrocatalytic Reduction of CO_2_ to Paraffin Using Pn Heterojunctions,” iScience 23 (2020): 100768. 31887657 10.1016/j.isci.2019.100768 PMC6941872

[85] J. Luo , L. Steier , M. K. Son , M. Schreier , M. T. Mayer , and M. Grätzel , “Cu_2_O Nanowire Photocathodes for Efficient and Durable Solar Water Splitting,” Nano Letters 16 (2016): 1848. 26866762 10.1021/acs.nanolett.5b04929

[86] M. A. Rahman , J. P. Thomas , and K. T. Leung , “A Delaminated Defect‐Rich ZrO_2_ Hierarchical Nanowire Photocathode for Efficient Photoelectrochemical Hydrogen Evolution,” Advanced Energy Materials 8 (2018): 1701234.

[87] C. A. Bignozzi , S. Caramori , V. Cristino , R. Argazzi , L. Meda , and A. Tacca , “Nanostructured Photoelectrodes Based on WO_3_: Applications to Photooxidation of Aqueous Electrolytes,” Chemical Society Reviews 42 (2013): 2228. 23223715 10.1039/c2cs35373c

[88] J. Pan , G. Liu , G. Q. Lu , and H. M. Cheng , “On the True Photoreactivity Order of {001}, {010}, and {101} Facets of Anatase TiO_2_ Crystals,” Angewandte Chemie International Edition 50 (2011): 2133. 21344568 10.1002/anie.201006057

[89] G. Liu , L. C. Yin , J. Pan , et al., “Greatly Enhanced Electronic Conduction and Lithium Storage of Faceted TiO_2_ Crystals Supported on Metallic Substrates by Tuning Crystallographic Orientation of TiO_2_ ,” Advanced Materials 27 (2015): 3507. 25939878 10.1002/adma.201500198

[90] S. Selcuk and A. Selloni , “Facet‐dependent Trapping and Dynamics of Excess Electrons at Anatase TiO_2_ Surfaces and Aqueous Interfaces,” Nature Materials 15 (2016): 1107. 27322821 10.1038/nmat4672

[91] M. Zhao , H. Xu , H. Chen , et al., “Photocatalytic Reactivity of {121} and {211} Facets of Brookite TiO_2_ Crystals,” Journal of Materials Chemistry A 3 (2015): 2331.

[92] Y. Li , X. Yun , H. Chen , W. Zhang , and Y. Li , “Facet‐Selective Charge Carrier Transport, Deactivation Mechanism and Stabilization of a Cu_2_O Photo‐Electro‐Catalyst,” Physical Chemistry Chemical Physics 18 (2016): 7023. 26898270 10.1039/c6cp00297h

[93] N. Li , M. Liu , Z. Zhou , J. Zhou , Y. Sun , and L. Guo , “Charge Separation in Facet‐engineered Chalcogenide Photocatalyst: A Selective Photocorrosion Approach,” Nanoscale 6 (2014): 9695. 24993804 10.1039/c4nr02068e

[94] G. Liu , P. R. Narangari , Q. T. Trinh , et al., “Manipulating Intermediates at the Au–TiO_2_ Interface Over InP Nanopillar Array for Photoelectrochemical CO_2_ Reduction,” ACS Catalysis 11 (2021): 11416.

[95] J. Hu , N. Fan , C. Chen , et al., “Facet Engineering in Au Nanoparticles Buried in p‐Si Photocathodes for Enhanced Photoelectrochemical CO_2_ Reduction,” Applied Catalysis B 327 (2023): 122438.

[96] S. Mubarak , D. Dhamodharan , H. S. Byun , S. B. Arya , and D. K. Pattanayak , “Effective Photoelectrocatalytic Reduction of CO_2_ to Formic Acid Using Controllably Annealed TiO2 Nanoparticles Derived From Porous Structured Ti Foil,” Journal of CO2 Utilization 63 (2022): 102152.

[97] B. Paul , N. Manwar , P. Bhanja , et al., “Morphology Controlled Synthesis of 2D Heterostructure Ag/WO_3_ Nanocomposites for Enhanced Photoelectrochemical CO_2_ Reduction Performance,” Journal of CO2 Utilization 41 (2020): 101284.

[98] J. W. Beeman , J. Bullock , H. Wang , et al., “Si Photocathode With Ag‑Supported Dendritic Cu Catalyst for CO_2_ Reduction,” Energy & Environmental Science 12 (2019): 1068.

[99] P. A. Kempler , M. H. Richter , W. H. Cheng , B. S. Brunschwig , and N. S. Lewis , “Si Microwire‐Array Photocathodes Decorated With Cu Allow CO_2_ Reduction With Minimal Parasitic Absorption of Sunlight,” ACS Energy Letters 5 (2020): 2528.

[100] J. C. Wu , J. Zheng , P. Wu , and R. Xu , “Study of Native Defects and Transition‐Metal (Mn, Fe, Co, and Ni) Doping in a Zinc‐Blende CdS Photocatalyst by DFT and Hybrid DFT Calculations,” Journal of Physical Chemistry C 115 (2011): 5675.

[101] B. Zhang , L. Wang , Y. Zhang , Y. Ding , and Y. Bi , “Ultrathin FeOOH Nanolayers With Abundant Oxygen Vacancies on BiVO_4_ Photoanodes for Efficient Water Oxidation,” Angewandte Chemie International Edition 57 (2018): 2248. 29333765 10.1002/anie.201712499

[102] D. Liu , Y. Lv , M. Zhang , et al., “Defect‐related Photoluminescence and Photocatalytic Properties of Porous ZnO Nanosheets,” Journal of Materials Chemistry A 2 (2014): 15377.

[103] H. Wu , F. Meng , L. Li , S. Jin , and G. Zheng , “Dislocation‐Driven CdS and CdSe Nanowire Growth,” ACS Nano 6 (2012): 4461. 22519752 10.1021/nn301194v

[104] S. W. Lee , S. Chen , W. Sheng , et al., “Roles of Surface Steps on Pt Nanoparticles in Electro‐oxidation of Carbon Monoxide and Methanol,” Journal of the American Chemical Society 131 (2009): 15669. 19824642 10.1021/ja9025648

[105] J. Yan , G. Wu , N. Guan , L. Li , Z. Li , and X. Cao , “Understanding the Effect of Surface/Bulk Defects on the Photocatalytic Activity of TiO_2_: Anatase versus Rutile,” Physical Chemistry Chemical Physics 15 (2013): 10978. 23708180 10.1039/c3cp50927c

[106] M. Kong , Y. Li , X. Chen , et al., “Tuning the Relative Concentration Ratio of Bulk Defects to Surface Defects in TiO_2_ Nanocrystals Leads to High Photocatalytic Efficiency,” Journal of the American Chemical Society 133 (2011): 16414. 21923140 10.1021/ja207826q

[107] R. Qu , W. Zhang , N. Liu , et al., “Antioil Ag_3_PO_4_ Nanoparticle/Polydopamine/Al_2_O_3_ Sandwich Structure for Complex Wastewater Treatment: Dynamic Catalysis Under Natural Light,” ACS Sustainable Chemistry & Engineering 6 (2018): 8019.

[108] Y. Yang , L. C. Yin , Y. Gong , et al., “An Unusual Strong Visible‐Light Absorption Band in Red Anatase TiO_2_ Photocatalyst Induced by Atomic Hydrogen‐Occupied Oxygen Vacancies,” Advanced Materials 30 (2018): 1704479. 10.1002/adma.201704479 29315852

[109] J. Yan , T. Wang , G. Wu , et al., “Tungsten Oxide Single Crystal Nanosheets for Enhanced Multichannel Solar Light Harvesting,” Advanced Materials 27 (2015): 1580. 25582656 10.1002/adma.201404792

[110] W. J. Dong , J. W. Lim , D. M. Hong , et al., “Grain Boundary Engineering of Cu–Ag Thin‐Film Catalysts for Selective (Photo)Electrochemical CO_2_ Reduction to CO and CH_4_ ,” ACS Applied Materials & Interfaces 13 (2021): 18905. 33848138 10.1021/acsami.1c03735

[111] J. Cheng , X. Yang , X. Xuan , N. Liu , and J. Zhou , “Development of an Efficient Catalyst With Controlled Sulfur Vacancies and High Pyridine Nitrogen Content for the Photoelectrochemical Reduction of CO_2_ Into Methanol,” Science of the Total Environment 702 (2020): 134981. 31715395 10.1016/j.scitotenv.2019.134981

[112] M. Kan , C. Yang , Q. Wang , et al., “Defect‐Assisted Electron Tunneling for Photoelectrochemical CO_2_ Reduction to Ethanol at Low Overpotentials,” Advanced Energy Materials 12 (2022): 2201134.

[113] S. Zhou , K. Sun , J. Huang , et al., “Accelerating Electron‐Transfer and Tuning Product Selectivity through Surficial Vacancy Engineering on CZTS/CdS for Photoelectrochemical CO_2_ Reduction,” Small 17 (2021): 2100496. 10.1002/smll.202100496 34173332

